# In Vitro and In Silico Investigation of Polyacetylenes from *Launaea capitata* (Spreng.) Dandy as Potential COX-2, 5-LOX, and BchE Inhibitors

**DOI:** 10.3390/molecules28083526

**Published:** 2023-04-17

**Authors:** Fatma M. Abdel Bar, Amira Mira, Ahmed I. Foudah, Manal A. Alossaimi, Shatha F. Alkanhal, Alanoud M. Aldaej, Mai H. ElNaggar

**Affiliations:** 1 Department of Pharmacognosy, College of Pharmacy, Prince Sattam bin Abdulaziz University, Al-Kharj 11942, Saudi Arabia; a.foudah@psau.edu.sa; 2 Faculty of Pharmacy, Mansoura University, Mansoura 35516, Egypt; 3 Department of Pharmacognosy & Pharmaceutical Chemistry, College of Dentistry & Pharmacy, Buraydah Private Colleges, Buraydah 51418, Saudi Arabia; Amira.Mera@bpc.edu.sa; 4 Department of Pharmacognosy, Faculty of Pharmacy, Mansoura University, Mansoura 35516, Egypt; 5 Department of Pharmaceutical Chemistry, College of Pharmacy, Prince Sattam bin Abdulaziz University, Al-Kharj 11942, Saudi Arabia; 6 College of Pharmacy, Prince Sattam bin Abdulaziz University, Al-Kharj 11942, Saudi Arabia; 7 Department of Pharmacognosy, Faculty of Pharmacy, Kafrelsheikh University, Kafrelsheikh 33516, Egypt

**Keywords:** polyacetylene glycosides, *Launaea capitata*, butyrylcholinesterase, 5-lipoxygenase, cyclooxygenase-2, neuroinflammation

## Abstract

Diverse secondary metabolites are biosynthesized by plants via various enzymatic cascades. These have the capacity to interact with various human receptors, particularly enzymes implicated in the etiology of several diseases. The *n*-hexane fraction of the whole plant extract of the wild edible plant, *Launaea capitata* (Spreng.) Dandy was purified by column chromatography. Five polyacetylene derivatives were identified, including (3*S*,8*E*)-deca-8-en-4,6-diyne-1,3-diol (**1A**), (3*S*)-deca-4,6,8-triyne-1,3-diol (**1B**), (3*S*)-(6*E*,12*E*)-tetradecadiene-8,10-diyne-1,3-diol (**2**), bidensyneoside (**3**), and (3*S*)-(6*E*,12*E*)-tetradecadiene-8,10-diyne-1-ol-3-*O*-β-D-glucopyranoside (**4**). These compounds were investigated for their in vitro inhibitory activity against enzymes involved in neuroinflammatory disorders, including cyclooxygenase-2 (COX-2), 5-lipoxygenase (5-LOX), and butyrylcholinesterase (BchE) enzymes. All isolates recorded weak–moderate activities against COX-2. However, the polyacetylene glycoside (**4**) showed dual inhibition against BchE (IC_50_ 14.77 ± 1.55 μM) and 5-LOX (IC_50_ 34.59 ± 4.26 μM). Molecular docking experiments were conducted to explain these results, which showed that compound **4** exhibited greater binding affinity to 5-LOX (−8.132 kcal/mol) compared to the cocrystallized ligand (−6.218 kcal/mol). Similarly, **4** showed a good binding affinity to BchE (−7.305 kcal/mol), which was comparable to the cocrystallized ligand (−8.049 kcal/mol). Simultaneous docking was used to study the combinatorial affinity of the unresolved mixture **1A**/**1B** to the active sites of the tested enzymes. Generally, the individual molecules showed lower docking scores against all the investigated targets compared to their combination, which was consistent with the in vitro results. This study demonstrated that the presence of a sugar moiety (in **3** and **4**) resulted in dual inhibition of 5-LOX and BchE enzymes compared to their free polyacetylenes analogs. Thus, polyacetylene glycosides could be suggested as potential leads for developing new inhibitors against the enzymes involved in neuroinflammation.

## 1. Introduction

Nature remains a crucial resource for drug leads and has a considerable contribution to the field of drug discovery [1]. Many medicinal plants were reported to have beneficial effects on patients with Alzheimer’s disease (AD), such as *Gingko biloba*, *Bacopa monnieri*, *Salvia officinalis*, *Melissa officinalis*, *Withania somnifera*, *Centella asiatica*, *Tinospora cordifolia*, *Curcuma longa*, and *Glycyrrhiza glabra* [2,3,4]. It was demonstrated that the efficacy of these plants in the treatment of AD is due to several phytochemical classes, such as polyphenols, flavonoids, triterpenoids, steroids, and alkaloids [2,4]. Moreover, several mechanisms were found to contribute to the anti-AD action of these phytochemicals, involving antioxidant, anticholinesterase, anti-neuroinflammatory, anti-amyloid, and anti-hyperphosphorylation of the Tau protein [5,6]. In the past, researchers have focused on the amyloid plaques and neurofibrillary tangles theory as the most influential etiological factor for Alzheimer’s disease (AD). Hence, most drug-discovery studies focused on the cholinergic theory to treat AD. Recently, it was found that the generated *β*-amyloid interacts with receptors expressed by active microglia and triggers the production of inflammatory cytokines [7]. Furthermore, inflammatory markers are key indicators in the involvement of immune activation [8]. In addition, the hyperactivated glial cell releases several toxic products, including reactive oxygen species (ROS), nitric oxide (NO), and proteolytic enzymes, which cause damage to neurons and eventually cause death [3]. Thus, targeting neuroinflammation could be an additional strategy alongside the cholinergic theory in the treatment of AD.

By comprising about 1600 to 1700 genera, the Asteraceae family is considered the largest known plant family with great botanical, phytochemical, and geographical diversity [9,10,11]. The diverse classes of bioactive secondary metabolites of the Aster family significantly contribute to many success stories in the field of drug discovery and therapeutic applications [10]. The genus *Launaea* Cass. (Asteraceae) encompasses several traditional plants, which are used by many cultures as appetite stimulants, digestives, antidiarrheals, anti-inflammatories, galactagogues, hepatoprotectives, sedatives, diuretics, and antipyretics, in addition to their use in wound healing [12]. Members of the genus *Launaea* were reported to contain flavonoids, polyacetylenes, lignans, coumarins, quinic acid derivatives, galactolipids, sesqui-, and tri-terpenoids [12,13,14,15,16,17,18]. To date, the phytochemical and biological studies on this genus are still limited [16,17].

*Launaea capitata* is a wild edible Saudi plant, which is commonly known by native people as “Al-baqraa, Al-buqair, or Hwaa Al-baqar”. Our research group reported the isolation of new galactolipids from this plant and investigated their COX-2, 5-LOX, and BchE inhibitory activities [18]. In this study, as a continuation of the efforts to explore its chemical composition*,* the *n*-hexane fraction of the methanol extract of *L. capitata* whole plant was explored using column chromatographic techniques. The isolates were identified by several spectroscopic methods, involving one- and two-dimensional NMR and high-resolution mass spectrometry. The isolated compounds were assessed for their in vitro inhibitory activities against three suggested enzymes as potential targets for neuroinflammatory disorders associated with AD, including COX-2, 5-LOX, and BchE. Additionally, a molecular docking study was carried out to explain the in vitro inhibitory activity, understand the mode of binding, and figure out the presence of crucial interactions between the investigated structures and the active sites of these enzymes.

## 2. Results and Discussion

### 2.1. Identification of the Isolated Compounds

#### 2.1.1. Identification of Compound **1A** and **1B**

**Figure 1:**
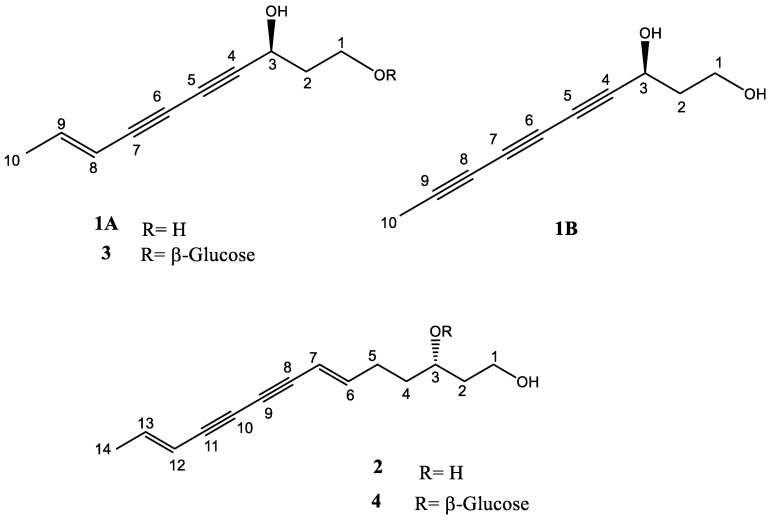
Structures of isolated compounds (**1A**/**1B**, **2**–**4**) from *Launaea capitata*.

The spectral data of **1** indicated that it was isolated as a non-resolved mixture of two compounds; **1A** and **1B** (ratio about 100:60, respectively), Figure 1. The two compounds couldn’t be separated because of the small amount (5 mg) of the obtained mixture. However, the simplicity of the two structures, allowed for their unambiguous identification. The ^1^H NMR, DEPT135, and HSQC spectra of **1** (Table 1 and Figures S1, S3 and S5) showed the presence of two olefinic proton signals, each was integrated for one proton at *δ*_H_ 5.59 (d, *J* = 15.8 Hz, H-8A; *δ*_C_ 110.5) and 6.31 (m, H-9A; *δ*_C_ 145.1). A methyl proton signal at *δ*_H_ 1.82 (dd, *J* = 6.9, 1.6 Hz, H-10A; *δ*_C_ 18.9) was found to be attached to the olefinic carbon (C-9A), as revealed from the HMBC spectrum (Figure S6), which showed correlations for this methyl group with C-8A and C-9A. The DEPT135 spectrum (Figure S3) showed three aliphatic carbon signals distinguished as; two methylenes at *δ*_C_ 59.1 (C-1A; *δ*_H_ 3.66, m, overlapped) and 41.3 (C-2A; *δ*_H_ 1.86, m), and a methine carbon signal at *δ*_C_ 60.3 (C-3A; *δ*_H_ 4.58, t, *J* = 6.8 Hz). The HMBC spectrum showed correlations of H-1A/C-2A, H-3A/C-1A, and H-3A/C-2A confirming their connectivity. Furthermore, it showed HMBC correlations for the proton H-3A with the quaternary carbon signals at *δ*_C_ 83.5 (C-4A), 69.7 (C-5A), and 72.4 (C-6A), and for the proton H-9A with the quaternary carbon signal at *δ*_C_ 78.1 (C-7A), thereby confirming the presence of a central diyne moiety of a polyacetylene derivative. The HR-MS (positive mode) spectrum of compound **1** (Figure S7) showed a pseudo molecular ion peak for **1A** at m/z 187.0728 for [M + Na]^+^ (Calcd. 187.0730), which agreed with the molecular formula, C_10_H_12_O_2_. By reviewing the data with those published in the literature, compound **1A** was confirmed to be the known polyacetylene derivative, (8*E*)-deca-8-en-4,6-diyne-1,3-diol isolated before from *Gymnaster koraiensis* (Asteraceae) [19,20]. However, it is worth noting that in their first report, Jung et al. [20] reported the absolute configuration of this compound as (3*R*). However, the same research group later published the acyl-CoA cholesterol acyltransferase (ACAT) inhibitory activity of the same compound, although they assigned (3*S*) for its absolute configuration by referring to the same spectral data in their previously published paper [19]. Thus, we compared **1A** to its C_1_-*O*-glycoside, namely bidensyneoside A1 (**3**), which was isolated before from *L. capitata* [16]. Since it was concluded by the last research group that all compounds isolated from *L. capitata* had an *S* configuration [16], compound **1A** was biosynthetically suggested as (3*S*,8*E*)-deca-8-en-4,6-diyne-1,3-diol, the aglycone of bidensyneoside A1 [16,21].

The second compound of the mixture (**1B**) was closely related to **1A** with minor differences. Compound **1B** showed the absence of the olefinic bond as it was found to be replaced by an additional acetylene group and appeared to be a biosynthetic product of the dehydrogenation of **1A** [22]. This was evident from the ^1^H NMR, DEPT135, and HSQC spectra of **1** (Table 1 and Figures S1, S3 and S5), which showed a highly shielded methyl group at *δ*_C_ 3.81 (C-10B; *δ*_H;_ 1.99, s). This methyl group showed HMBC correlation with two quaternary carbon signals at 64.5 (C-8B), and 78.0 (C-9B) confirming the presence of an additional acetylene group at C-8B. The HR-MS (positive mode) spectrum of compound **1** (Figure S7) showed a pseudo molecular ion peak for **1B** at m/z 167.0126 for [M-H_2_O + Na]^+^ (Calcd. 167.0473), which agreed with a molecular formula of C_10_H_10_O_2_. Thus, compound **1B** was confirmed as (3*S*)-deca-4,6,8-triyne-1,3-diol, which was reported before in the Asteraceae family in *Artemisia capillaris* [23] and *Lactuca sativa* [24]. It could be concluded that the C_1_-*O*-glucosides of both **1A** and **1B** were previously reported from the Asteraceae plant, *Bidens parviflora* WILLD. and were termed bidensyneoside A1 and bidensyneoside B, respectively [21]. However, their aglycones (**1A,** and its biosynthetic dehydrogenated product, **1B**) were reported herein for the first time from *L. capitata*.

#### 2.1.2. Identification of Compound **2**

The ^1^H NMR, DEPT135, and HSQC spectra of **2** (Table 2 and Figures S8, S10 and S12) displayed two *trans* olefinic bonds that appeared as two signal groups; the first group comprised two overlapped proton doublets at *δ*_H_ 5.63 (d, *J* = 15.7 Hz, H-7; *δ*_C_ 109.2) and 5.61 (d, *J* = 15.7 Hz, H-12; *δ*_C_ 109.2), whereas the second downfield group included two proton multiplets resonating at *δ*_H_ 6.30 (m, H-6; *δ*_C_ 149.2) and 6.28 (m, H-13; *δ*_C_ 144.5). The presence of an attached saturated chain formed of five carbons from C-1 to C-5 was evident from the DEPT135 and HSQC experiments (Figures S10 and S12), which showed four methylene carbon signals at *δ*_C_ 60.1 (C-1; *δ*_H_ 3.70), 40.8 (C-2; *δ*_H_ 1.61), 37.5 (C-4; *δ*_H_ 1.51), and 30.5 (C-5; *δ*_H_ 2.20 & 2.28) and an oxymethine at 69.1 (C-3; *δ*_H_ 3.67). The ^1^H–^1^H COSY spectrum (Figure S11) correlated with the protons H_2_-1/H_2_-2, H_2_-2/H-3, H-3/H_2_-4, and H_2_-4/H_2_-5, confirming the sequence of their attachment. This was proven by the HMBC spectrum (Figure S13) that revealed correlations between H-2/C-1, H-3/C-2, H-4/C-3, H-5/C-4, H-5/C-6, H-5/C-7, H-6/C-4, and H-7/C-5. The ^13^C NMR spectrum (Table 2 and Figure S9) showed four quaternary carbon signals at 80.4 (C-8), 73.1 (C-9), 73.4 (C-10), and 80.3 (C-11), which suggested the presence of a middle conjugated diyne system. This was proven by the HMBC spectrum that showed significant correlations of H-6/C-8, H-7/C-9, H-14/C-13, H-14/C-12, H-12/C-11, and C-10. The HR-MS (positive mode) spectrum of compound **2** (Figure S14) showed an m/z at 219.1377 of [M + Na]^+^ (Calcd. at 219.1385), which agreed with the molecular formula, C_14_H_18_O_2_. By reviewing the spectral data of similar compounds, the structure of **2** was confirmed as (3*S*)-(6*E*,12*E*)-tetradecadiene-8,10-diyne-1,3-diol, which had been isolated before in several Asteraceae plants, including *Carthamus tinctorius* [25,26], *Coreopsis tinctoria* [27], *Echinops ritro* [28], and *Atractylodes lancea* [29]. The “S” configuration was biosynthetically suggested for C-3 since compound **2** was confirmed as the aglycone of the C_1_-*O*-glucoside, namely bidensyneoside E, previously isolated from *L. capitata* [16]. However, this study described the first isolation of the aglycone derivative of this compound from *L. capitata*.

#### 2.1.3. Identification of Compound **3**

The ^1^H NMR, DEPT135, and HSQC spectra of **3** (Table 2 and Figures S15, S17 and S19) showed the presence of two olefinic proton signals at *δ*_H_ 5.58 (d, *J* = 18.5, H-8; *δ*_C_ 110.2) and 6.32 (dd, *J* = 15.7 and 6.3 Hz, H-9; *δ*_C_ 145.2), and a methyl proton doublet at *δ*_H_ 1.82 (*J* = 6.3 Hz, H_3_-10; *δ*_C_ 19.0), which suggested the presence of a *trans* double bond with a methyl substitution at C-9. They also confirmed the presence of a β-linked glucosyl moiety from the carbon signals at *δ*_C_ 104.1 (C-1`; *δ*_H_ 4.30), and 74.7 (C-2`; *δ*_H_ 3.19), 77.6 (C-3`; *δ*_H_ 3.40), 71.1 (C-4`; *δ*_H_ 3.34), 77.4 (C-5`; *δ*_H_ 3.30), 62.3 (C-6`; *δ*_H_ 3.87 & 3.70). Moreover, they showed the presence of three additional protonated carbon signals, including two methylene carbon signals at *δ*_C_ 66.6 (C-1; *δ*_H_ 3.99 & 3.72) and 38.6 (C-2; *δ*_H_ 1.79), and a methine at *δ*_C_ 59.9 (C-3; *δ*_H_ 4.66). The ^13^C NMR spectrum of **3** showed the presence of four quaternary carbon signals at *δ*_C_ 83.5, 69.9, 72.3, and 78.1 assigned to a central conjugated diyne moiety at C-4 to C-7. This was confirmed from the HMBC spectrum (Figure S20) that showed correlations of H-3 with C-4 (*δ*_C_ 83.5), C-5 (*δ*_C_ 69.6), C-6 (*δ*_C_ 72.3), C-7 (*δ*_C_ 78.1). Additionally, HMBC correlations were found between H-8 and C-6, C-9, and C-10. The presence of an HMBC correlation between H-1` and C-1 confirmed the position of the glycosidic linkage. Moreover, the HR-MS (positive mode) spectrum of compound **3** (Figure S21) showed an m/z at 349.1244 of [M + Na]^+^ (Calcd. at 349.1263), which agreed with the molecular formula, C_16_H_22_O_7_. Thus, compound **1A** was identified as the aglycone of compound **3**. Consequently, the structure of **3** was confirmed to be the known polyacetylene structure, (3*S*,8*E*)-deca-8-en-4,6-diyne-1,3-diol-3-*O*-β-D-glucopyranoside, namely bidensyneoside A1, which was reported before from the aerial parts of *L. capitata* [16].

#### 2.1.4. Identification of Compound **4**

The spectral data of **4** (Figures S22–S28) were closely related to that of **3,** except for the presence of a C_14_ polyacetylene chain and appeared to be the 3-β-*O*-glucosyl derivative of compound **2**. This was revealed from ^1^H NMR, DEPT135, and HSQC (Table 2 and Figures S22, S24 and S26) and showed the presence of a β-glucosyl moiety and two *trans* double bonds at *δ*_C_ 149.7 (C-6; *δ*_H_ 6.32, m), 109.8 (C-7; *δ*_H_ 5.64, d, *J* = 16.7 Hz), 110.9 (C-12; *δ*_H_ 5.61, d, *J* = 16.7 Hz), and 144.4 (C-13; *δ*_H_ 6.28, m). In addition, they showed the presence of a segment formed of five saturated carbon atoms compared to three carbons in the case of **3**. This was evident from four methylene carbon signals at *δ*_C_ 59.5 (C-1; *δ*_H_ 3.66 & 3.76), 38.2 (C-2; *δ*_H_ 1.75), 35.4 (C-4; *δ*_H_ 1.71 & 1.65), 30.1 (C-5; *δ*_H_ 2.30), and a downfield methine at *δ*_C_ 77.9 (C-3; *δ*_H_ 3.89). The ^1^H–^1^H COSY spectrum (Figure S25) showed correlations with the protons H_2_-1/H_2_-2, H_2_-2/H-3, H-3/H_2_-4, and H_2_-4/H_2_-5 confirming the sequence of their attachment. The HMBC spectrum showed correlations of H-5 with C-3, C-4, C-6, and C-7 (Figure S27). The ^13^C NMR spectrum of **4** (Figure S23) revealed the presence of four quaternary carbons in a diyne system at *δ*_C_ 80.6 (C-8), 73.2 (C-9), 73.3 (C-10), and 80.3 (C-11). The presence of this diyne moiety was further confirmed by the HMBC correlations to H-6/C-8, H-7/C-9, H-12/C-10, H-13/C-11, and H-12/C-10. Finally, the HR-MS spectrum (positive mode) of compound **4** (Figure S28) confirmed a molecular formula of C_20_H_28_O_7_ based on the pseudo molecular ion peak of [M + Na]^+^ at 403.1719 (Calcd. 403.1722). Therefore, the structure of compound **4** was confirmed as (3*S*)-(6*E*,12*E*)-tetradecadiene-8,10-diyne-1-ol-3-*O*-β-D-glucopyranoside, which was previously reported from *Coreopsis tinctoria* (Asteraceae) [27]. It is worth noting that compound **4** was confirmed as the C_3_-*O*-glucoside of **2**, isolated, herein, for the first time from *L. capitata*. However, its C_1_-*O*-glucoside derivative, termed bidensyneoside E, was previously reported in the same plant [16]. These findings supported the presence of the same configuration at C-3 for the three isolated plyacetylene derivatives from *L. capitata*.

### 2.2. In Vitro Enzyme Inhibition Assays

Generally, the results of the in vitro enzyme assays (Table 3) demonstrated that the investigated polyacetylenes (**1A**, **1B**, **2**–**4**) showed weak to moderate inhibitory activities against the COX-2 enzyme compared to the positive control, nordihydroguairetic acid (NDGA, IC_50_ 4.70 ± 0.76 μM). Compound **4** showed weak COX-2 inhibitory activity (146.38 ± 7.70 μM), followed by the polyacetylene mixture **1A**/**1B** (IC_50_ 170.48 ± 20.15 μM). However, compounds **2** and **3** were inactive at test conditions. Nevertheless, the investigated compounds showed a slightly better in vitro inhibition against 5-LOX compared to their effect on COX-2. Particularly, compound **4** showed moderate 5-LOX inhibitory activity with an IC_50_ of 34.59 ± 4.26 μM, compared to NDGA (IC_50_ 5.65 ± 0.89 μM). For the BchE enzyme, compound **4** similarly showed the greatest inhibitory activity (IC_50_ 14.77 ± 1.55 μM), which was comparable to rivastigmine (IC_50_ 14.06 ± 1.48 μM). Meanwhile, donepezil showed greater BchE inhibition with an IC_50_ of 5.77 ± 0.61 μM. This was followed by **3** (IC_50_ 48.81 ± 6.34 μM) and the polyacetylene mixture **1A**/**1B** (IC_50_ 58.60 ± 5.21 μM), which exhibited moderate BchE inhibitory activity.

By comparing the obtained results of COX-2 and 5-LOX with those published in the literature, it was found that the feruyol polyacetylene ester, (3*Z*,5*E*,11*E*)-tridecatriene-7,9-diynyl-1-*O*-(*E*)-ferulate from *Atractylodes lancea* rhizomes recorded potent inhibitory activities against 5-LOX (3.4 μM) and COX-1 (1.1 μM) [30]. Whereas other investigated aliphatic polyacetylene esters by the same study, including (4*E*,6*E*,12*E*)-tetradecatriene-8,10-dyne-1,3-dilyl-diacetate and *erythro*-(1,3*Z*,11*E*)-tridecatriene-7,9-diyne-5,6-diyl diacetate, showed moderate to weak activities against the same enzymes with IC_50_ values range from 46.3 to >200 μM, which were almost comparable to our obtained results [30]. In conclusion, polyacetylenes could be suggested as a promising scaffold for preparing new COXs and LOXs inhibitors possibly by introducing aromatic esters to their basic skeleton. Regarding the reported BchE activity, a comparison of the obtained results with the previously published studies indicated that polyacetylene glycosides (as in the case of compounds **3** and **4**) were more active as BchE inhibitors than their aglycones [31].

### 2.3. Docking Study

**Figure 2:**
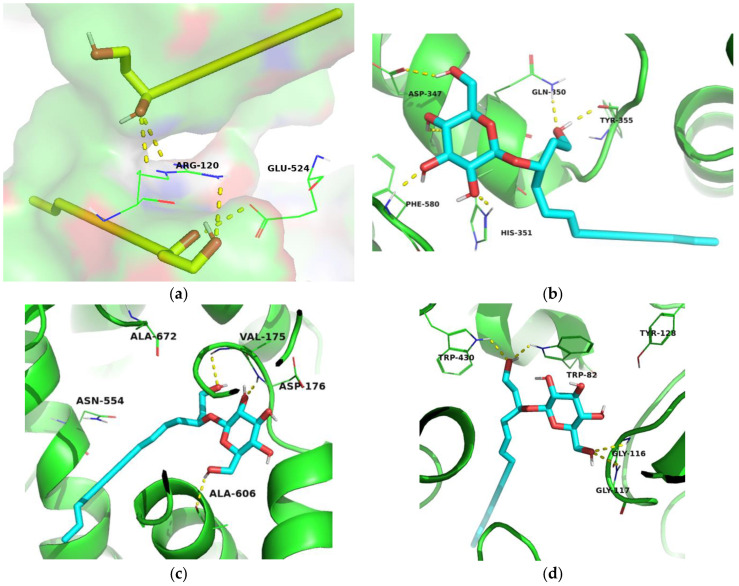
Three-dimensional molecular binding models of (**a**) Simultaneously docked molecules **1A** and **1B** (colored yellow) to the active site of COX-2; (**b**) docked molecule **4** (colored cyan) to the active site of COX-2; (**c**) docked molecule **4** (colored cyan) to the active site of 5-LOX; (**d**) docked molecule **4** (colored cyan) to the active site of BchE.

**Figure 3:**
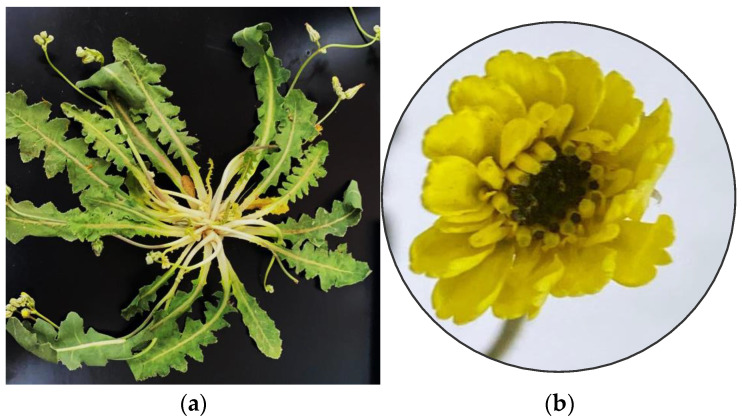
Photograph of *Launaea capitata* (Spreng.) Dandy; (**a**) whole plant, (**b**) the flower.

Molecular docking is a broadly used computational tool in drug discovery. It is very helpful for predicting the mode of interaction and the binding affinity of the ligands towards the investigated proteins [32,33]. Molecular docking was performed to explain the observed results of the in vitro enzyme inhibition assays and to investigate the mode of interaction of the tested molecules. The observed H-bonding of the tested compounds with the amino acid residues in the active sites of the investigated proteins are listed in Table S2. It is worth noting that compounds **1A** and **1B** were tested as a mixture in the in vitro enzyme assays, which is why the simultaneous docking function that permits the docking of multiple ligands to the same target was used to study their interaction with the investigated proteins. Interestingly, the use of this function has enabled us to explain the in vitro enzyme inhibition assay results. The tested **1A** and **1B** individual molecules showed low docking scores against all the investigated targets (Table 4). However, the tested **1A**/**1B** combination showed a reasonable docking score (−7.861 kcal/mol) against COX-2 enzyme, which was consistent with its slightly better in vitro inhibitory activity among the tested molecules with IC_50_ of 170.48 ± 20.15 μM (Table 3). Visualization of the best docking pose showed that the alcoholic groups of the two compounds formed H-bonds with Arg-120 (Figure 2a) located in the opening of the cyclooxygenase channel and is essential for COX-2 catalysis [34,35]. Compound **1A** also formed an H-bond with Glu-524 [34,35]. The cocrystallized ligand of COX-2 showed the highest docking score (−8.004 kcal/mol) and exhibited H-bonding interactions with Ser-530, and Tyr-385 amino acid residues essential for the enzyme activity (Table S2, Figure S29a) [35]. Compound 4 showed a reasonable docking score against COX-2 enzyme (−6.895 kcal/mol, Table 4) and showed the formation of H-bonding with Asp-347, Gln-350, His-351, Tyr-355, and phe-580 amino acid residues (Table S2, Figure 3b), which explain its highest in vitro inhibition activity against the COX-2 enzyme with an IC_50_ of 146.38 ± 7.70 μM.

Although the **1A**/**1B** combination showed the highest docking scores against 5-LOX and BchE, as shown by simultaneous docking (Table 4), no H-bonding interactions with key amino acids were observed during the visualization of their obtained docking poses against 5-LOX and BchE (Figures S29d and S29g, respectively). The natural substrate of 5-LOX and arachidonic acid was reported to form van der Waals contacts within the active site of 5-LOX, and no H-bonds were observed, as illustrated in Figure S29c [36]. While compound **4** showed H-bonding with the amino acid residues Val-175, Asp-176, and Ala-606 in the active site of 5-LOX (Figure 2c). Residues Val-175 and Asp-176 were reported to be a part of the V4 anchor of the 5-LOX’s active site and were suggested to be involved in the binding of novel 5-LOX inhibitors [36]. These obtained docking interactions together with the high docking score of compound **4** (−8.132 kcal/mol, Table 4) explained the highest in vitro inhibitory activity of this compound against 5-LOX. Compound **3** showed H-bonding interactions with His-372, His-367, and His-550 amino acid residues (Figure S29e) in the Fe coordination sphere of the 5-LOX active site explaining its in vitro enzyme inhibition activity [37].

Two molecules of the cocrystallized ligand were examined in the active site of BchE, as presented in Figure S29f. They were reported to form aromatic stacking with Tyr-332, although no H-bonds were reported to be observed [38]. Compound **4** showed H-bonding interactions with Trp-82, Gly-116, Gly-117, and Trp-430 amino acid residues in the active site of BchE (Figure 2d). Particularly, Gly116 and Gly117 amino acids were involved in the oxyanion hole in the active site of BchE [38]. Hence, the highest in vitro enzyme inhibition activity of compound **4** IC_50_ (14.77 ± 1.55 μM) could be attributed to its interaction with these residues. Compound **3** displayed three H-bonding interactions with Tyr-128, Trp-82, and His-438 residues (Figure S29h) in the active site of BchE [39,40]. It showed a lower docking score (−7.018 kcal/mol, Table 4) and a higher IC_50_ (48.81 ± 6.34 μM) than compound **4** against the BchE enzyme.

It can be concluded that the compound **4W** showed the best docking interactions against the investigated enzymes explaining its highest in vitro enzyme inhibition activity. The presence of the sugar moiety in the structures of compound **4** and compound **3** greatly enhanced their enzyme inhibitory activity in comparison to the free polyacetylene molecules, as in the case of compound **2**.

## 3. Materials and Methods

### 3.1. Plant Material

The whole plant of *Launaea capitata* (Spreng.) Dandy (Figure 3) was harvested in Riyadh, Saudi Arabia, in March 2022. A voucher sample (ID #16741) was kept at the herbarium of the Pharmacognosy Department, Prince Sattam bin Abdulaziz University, Al-Kharj. The identity of the plant was confirmed by Professor Ibrahim A. Mashaly, Department of Plant Ecology, Faculty of Sciences, Mansoura University, Egypt. The plant material was shade-dried, powdered, and kept for further phytochemical processing.

### 3.2. Chemicals and Instruments

Thin layer chromatographic analyses were performed using TLC silica gel, pore size 60 Å 60, F_254_ on aluminum sheets (Merck Millipore, Darmstadt, Germany). For column chromatographic analyses (CC.), various stationary phases were used, including silica gel, pore size 60 Å 60, mesh 70–230 (Merck Millipore, Darmstadt, Germany), and reversed-phase octadecyl (RP-C18) silica gel (Merck Millipore, Darmstadt, Germany). Various mobile phases were composed of reagent-grade solvents (Loba Chemie Pvt. Ltd., Mumbai, India). Then, 1- and 2-D NMR spectra were recorded on a Bruker UltraShield Plus 500 MHz spectrometer (Rheinstetten, Germany), with CD_3_OD as the solvent. Chemical shifts (*δ*) were obtained in part per million (ppm) and the coupling constants (*J*) were measured in hertz (Hz). Electrospray ionization mass spectrometry (ESI-MS) was obtained using a Thermo Scientific UPLC RS Ultimate 3000-Q Exactive hybrid quadrupole-Orbitrap mass spectrometer (Waltham, MA, USA).

### 3.3. Extraction and Purification

About 1200 g of the dry powder of the title plant was exhausted by maceration in cold MeOH (5 × 2000 mL). The combined methanolic extracts were evaporated at 45 °C by an R-215 rotavapor (Buchi, Switzerland). The obtained extract (325.0 g) was extracted by suspending it in water and shaking it with *n*-hexane (4 × 500 mL) to give a non-polar *n*-hexane fraction (87.3 g). Chromatographic separation of the obtained *n*-hexane fraction was carried out on a silica gel CC., (5 cm i.d. × 50 cm L.), packed in *n*-hexane, eluted with EtOAc (from 0→100%, gradient), then, by EtOAc-MeOH (0→100%, gradient), and the effluent volume was 250 mL. The obtained fractions (400) were monitored by normal phase silica gel and grouped based on their components to provide ten groups (Hex-I-X). Group Hex-IV (109–144, weight; 500 mg) eluted with *n*-hexane-EtOAc (80:20 and 75:25 *v*/*v*) was further chromatographed on a silica gel CC., (3 cm i.d. × 45 cm L.), packed in petroleum ether-CHCl_3_ (20:80 *v*/*v*), eluted using increasing proportions of CHCl_3_ (20:80→0:100 *v*/*v*), and the effluent was 100 mL. The obtained fractions were monitored by normal and reversed phase TLC. Fractions (35–58) eluted with ether-CHCl_3_ (10:90 *v*/*v*, weight; 120 mg), were further purified on an RP-C18 silica gel CC., (2 cm i.d. × 25 cm L.), packed in MeOH-H_2_O (70:30 *v*/*v*), eluted with methanol to 0:100 *v*/*v*, and an effluent volume of 5 mL. Fractions (3–4) eluted with MeOH-H_2_O (70:30 *v*/*v*) afforded compound **1** (5 mg), while fractions (5–6) eluted with MeOH-H_2_O (80:20 *v*/*v*) afforded compound **2** (4 mg). Both compounds showed faint quenching of UV_254_ light and orange-brown spots (0.69 and 0.62, respectively) using MeOH-H_2_O (60:40 *v*/*v*) and 10% sulfuric acid spray reagent. Group Hex-VIII (285–330, weight; 900 mg) eluted with EtOAc-MeOH (97:3 to 95:5 *v*/*v*) was further chromatographed on a silica gel CC., (3 cm i.d. × 45 cm L.), packed in CHCl_3_ and eluted with an increasing proportion of MeOH (100:0→70:30 *v*/*v*), and the effluent was 100 mL. Fractions (2–12) eluted with CHCl_3_-MeOH (95:5 *v*/*v*) were further purified on an RP-C18 silica gel CC., (2 cm i.d. × 25 cm L.), packed in MeOH-H_2_O (50:50 *v*/*v*), eluted with methanol to 0:100 *v*/*v*, and an effluent volume of 5 mL. Fractions (5–15) eluted with MeOH-H_2_O (50:50 *v*/*v*) afforded compound **3** (464 mg) and fractions (5–6) eluted with MeOH-H_2_O (80:20 *v*/*v*) afforded compound **4** (25.5 mg). They showed strong quenching of UV_254_ light and orange spots (0.56 and 0.22, respectively) using MeOH-H_2_O (60:40 *v*/*v*).

### 3.4. Enzyme Inhibition Assays

#### 3.4.1. BchE Inhibition Assay

The in vitro butyrylcholinesterase (BchE) inhibitory activity of the isolated polyacetylenes was determined according to the published method, using horse serum butyrylcholinesterase (Equine BchE; CAS#9001-08-5) with a slight change [18,41,42]. To create a useful stock solution, the materials were dissolved in DMSO. The maximal DMSO concentration was 0.1%, and twelve sample concentrations (ranging from 500 to 0.25 g/mL) were created by two-fold serial dilution in Tris buffer (pH 8.0) [18]. Positive controls included Rivastigmine (CAS# 123441-03-2, Sigma-Aldrich, St. Louis, MO, USA) and Donepezil (CAS# 120011-70-3, Sigma-Aldrich, St. Louis, MO, USA). The concentration of the test material that inhibited 50% of the BchE enzyme activity was represented by the concentration-response curve created by GraphPad Prism version 8.0 (San Diego, CA, USA), from which the IC_50_ values were derived.

#### 3.4.2. COX-2 Inhibition Assay

The fluorometric approach was used to measure the in vitro cyclooxygenase (COX-2) inhibitory activity. It was carried out in accordance with the COX-2 Inhibitor Screening Kit methodology, as advised by the manufacturer (CAT # K547-100, BioVision, Milpitas, CA, USA) [18,43,44]. The sample solutions were prepared in the manner described before providing twelve concentrations (ranging from 500 to 0.25 g/mL) [18]. The positive control was nordihydroguaiaretic acid (NDGA; CAS # 500-38-9, Sigma-Aldrich, St. Louis, MO, USA). The concentration-response curve created by GraphPad Prism version 8.0 (San Diego, CA, USA) was used to measure the IC_50_ (the concentration of the test sample that inhibited 50% of COX-2 enzyme activity).

#### 3.4.3. 5-LOX Inhibition Assay

The fluorometric approach was used to test the 5-LOX enzyme, in accordance with the manufacturer’s instructions, by using the enzyme’s inhibitor screening kit (CAT # K980-100, BioVision, Milpitas, CA, USA) to evaluate the 5-lipoxygenase (5-LOX) inhibitory activity in vitro [18,45,46]. Twelve concentrations (ranging from 500 to 0.25 g/mL) were prepared by serial dilution method [18]. The positive control was nordihydroguaiaretic acid (NDGA; CAS # 500-38-9, MO, Sigma-Aldrich, USA). The IC_50_ values were estimated from concentration-response curves generated by GraphPad Prism version 8.0 (San Diego, CA, USA), representing the concentration of the test sample that inhibited 50% of 5-LOX enzymatic activity.

### 3.5. Statistical Analysis

The IC_50_ values were represented as means (± SD) from triplicates of two independent experiments. The IC_50_ value was determined from the concentration-response curve processed using GraphPad Prism version 8.0 (San Diego, CA, USA), representing the concentration, which inhibited 50% of the enzyme activity.

### 3.6. Docking Study

Autodock vina 1.2.3 was used for performing the molecular docking study [47,48]. The crystal structures for COX-2, 5-LOX, and BchE were acquired from the RCSB protein data bank. Their PDB codes and the cocrystallized ligands are mentioned in Table S1. The structures of the proteins and ligands were prepared for the docking study with the help of Autodock tools. The docking parameters were recognized by selecting the coordinates of the binding sites using a grid box around the cocrystallized ligand with spacing of 0.375 Å. The dimensions of 30 × 30 × 30. The X, Y, and Z coordinates of the used grid box in each investigated protein are described in Table S1. The number of runs was determined by the exhaustiveness parameter and set to a value of 10, while the number of modes was set to 20. The docking process was validated by removing the cocrystallized ligand and redocking it into the active site using Autodock vina. Subsequently, the root mean square deviation (RMSD) value of the redocked ligand superimposed with the cocrystallized ligand was determined using Pymol and it was found to be lower than 2 Å. Docking poses showing minimum RMSD values were demonstrated using Pymol (version 2.5) [49].

## 4. Limitation of the Study

Although the active compounds showed inhibition of the target enzymes through endpoint inhibition reactions, determinations of the kinetic inhibition modes are essential to show their inhibition mechanisms. Furthermore, additional in vivo animal models are required to study the pharmacodynamics and pharmacokinetic properties of the active compounds.

## 5. Conclusions

In this study, as a continuation of our effort to discover the phytochemical composition of *Launaea capitata* (Spreng.) Dandy, the *n*-hexane fraction of the methanol extract of the whole plant was investigated. Four polyacetylene derivatives including three aglycones and one glycoside were isolated for the first time from this plant. in addition to a previously isolated glycoside derivative. The isolates showed reasonable 5-LOX and BchE inhibitory activities, however, they showed weak–moderate activities against COX-2. Docking experiments were conducted to clarify the mode of binding and to explain the results of the in vitro inhibitory activities. The current study suggested polyacetylene glycosides as promising leads for the dual inhibition of 5-LOX and BchE enzymes, which could be applied for the prevention and treatment of neuroinflammatory disorders, such as Alzheimer’s disease.

## Figures and Tables

**Table 1:** ^1^H (500 MHz, CD_3_OD) and ^13^C (125 MHz, CD_3_OD) NMR spectral data of compounds **1A** and **1B** (*J* in Hz).

C/H Position	1A		1B	
	^13^C	^1^H	^13^C	^1^H
**1**	59.1, CH_2_	3.66, m ^a^	58.9, CH_2_	3.66, m ^a^
**2**	41.3, CH_2_	1.86, m ^b^	41.2, CH_2_	1.86, m ^b^
**3**	60.2, CH	4.58, t (6.8) ^c^	60.1, CH	4.55, t (6.8) ^c^
**4**	83.5, C	---	79.2, C	---
**5**	69.70, C	---	69.7, C	---
**6**	72.4, C	---	64.9, C	---
**7**	78.1, C	---	59.0, C	---
**8**	110.5, CH	5.59, d (15.8)	64.5, C	---
**9**	145.1, CH	6.31, dq (6.9, 15.8)	78.0, C	---
**10**	18.9, CH_3_	1.82, dd (6.9, 1.6)	3.81, C	1.99, s

^a, b, c^ The same letters indicate overlapped signals.

**Table 2:** ^1^H (500 MHz, CD_3_OD) and ^13^C (125 MHz, CD_3_OD) NMR spectral data of compounds **2**–**4** (*J* in Hz).

C/H Position	2		3		4	
			^13^C	^1^H	^13^C	^1^H
**1**	60.1, CH_2_	3.70, t (6.3)	66.6, CH_2_	H_a_: 3.99, dd (10.0, 5.2) H_b_: 3.72, m	59.5, CH_2_	H_a_: 3.66, m H_b_: 3.76, m
**2**	40.8, CH_2_	1.61, m	38.6, CH_2_	1.79, brd (5.2)	38.2, CH_2_	1.75, m
**3**	69.1, CH	3.67, m	59.9, CH	4.66, t (6.2)	77.9, CH	3.89, m
**4**	37.5, CH_2_	1.51, m	83.5, C	---	35.4, CH_2_	H_a_: 1.71, m H_b_: 1.65, m
**5**	30.5, CH_2_	2.20, m 2.28, m	69.9, C	---	30.1, CH_2_	2.30, dd (14.5, 7.2)
**6**	149.2, CH	6.30, m ^b^	72.3, C	---	149.7, CH	6.32, m
**7**	109.2, CH	5.63, d (15.7) ^a^	78.1, C	---	109.8, CH	5.64, d (16.7)
**8**	80.4, C	---	110.2, CH	5.5, d (15.8)	80.6, C	---
**9**	73.1, C	---	145.2, CH	6.32, dd (15.7, 7.0)	73.2, C	---
**10**	73.4, C	---	19.0, CH_3_	1.82, d (6.3)	73.3, C	---
**11**	80.3, C	---			80.3, C	---
**12**	110.8, CH	5.61, d (15.7) ^a^			110.9, CH	5.61, d (16.7)
**13**	144.5, CH	6.28, m ^b^			144.4, CH	6.28, m
**14**	18.9, CH_3_	1.83, d (6.8)			18.9, CH_3_	1.82, d (6.8)
**1`**			104.1, CH	4.30, d (7.7)	103.9, CH	4.36, d (7.8)
**2`**			74.7, CH	3.19, t (8.2)	75.3, CH	3.16, m
**3`**			77.6, CH	3.40, m	78.1, CH	3.34, m
**4`**			71.1, CH	3.34, m	71.6, CH	3.22, m
**5`**			77.4, CH	3.30, m	77.7, CH	3.26, m
**6`**			62.3, CH_2_	H_a_: 3.87, d (11.5) H_b_: 3.70, m	62.7, CH_2_	H_a_: 3.87, m H_b_: 3.71, d (5.2)

^a, b^ The same superscript letters indicate interchangeable values.

**Table 3:** IC_50_ (μM) values of compounds **1A**/**1B** and **2**–**4** against COX-2, 5-LOX, and BchE enzymes in vitro.

Sample Code	COX-2	5-LOX	BchE
**1A/1B**	170.48 ± 20.15	>200	58.60 ± 5.21
**2**	>200	>200	179.02 ± 15.62
**3**	>200	80.96 ± 5.79	48.81 ± 6.34
**4**	146.38 ± 7.70	34.59 ± 4.26	14.77 ± 1.55
NDGA *	4.70 ± 0.76	5.65 ± 0.89	---
**Rivastigmine**	---	---	14.06 ± 1.48
**Donepezil**	---	---	5.77 ± 0.61

* Reference inhibitor: nordihydroguaiaretic acid (NDGA).

**Table 4:** Docking scores of compounds (**1A**/**1B** and **2**–**4**) against COX-2, 5-LOX, and BchE enzymes using AutoDock Vina 1.2.3.

Compound	Binding Energy (kcal/mol)		
	Cyclooxygenase-II (COX-2)	5-Lipooxygenase (5-LOX)	Butyrylcholinesterase (BchE)
**1A**	−5.263	−5.602	−5.399
**1B**	−5.179	−5.838	−5.17
**1A/1B (simultaneous docking)**	−7.861	−8.703	−8.549
**2**	−4.983	−6.422	−5.525
**3**	−6.987	−8.032	−7.018
**4**	−6.895	−8.132	−7.305
**Cocrystallized ligand**	−8.004	−6.218	−8.049

## Data Availability

Data sharing is not applicable to this article.

## Supplementary Materials

The following supporting information can be downloaded at: https://www.mdpi.com/article/10.3390/molecules28083526/s1, Figures S1–S28: 1H, ^13^C, DEPT135, ^1^H–^1^H COSY, HSQC, HMBC, and HR-MS spectra of isolated compounds **1**–**4**; Figure S29: 3D binding models of the cocrystallized ligand in the active site of COX-2; **3** in the active site of COX-2; **4** in the active site of COX-2; the cocrystallized ligand (arachidonic acid) in the active site of 5-LOX; simultaneously docked molecules **1A** and **1B** in the active site of 5-LOX; the cocrystallized ligand in the active site of BchE. Table S1: PDB codes of the crystal structures and grid box coordinates for the enzymes used in the docking study. Table S2: A list of H-bonding interactions of compounds (**1A**/**1B** and **3**–**4**) against COX-2, 5-LOX, and BchE enzymes compared to the interactions of the cocrystallized ligands.
