# Supplementary material for: In Vitro and In Silico Investigation of Polyacetylenes from Launaea capitata (Spreng.) Dandy as Potential COX-2, 5-LOX, and BchE Inhibitors
Source: Molecules. 2023 Apr 17;28(8):3526. doi: 10.3390/molecules28083526 (PMC10145610; doi:10.3390/molecules28083526)
Supplement: Supplementary file 1 [file molecules-28-03526-s001.zip › molecules-2332401-supplementary.pdf]

Supplementary Material

Table of Contents	Page
**Figure S1.** ^{1}H NMR spectrum of compound **1.**	3
**Figure S2.** ^{13}C NMR spectrum of compound **1**.	4
**Figure S3.** DEPT135 spectrum of compound **1**.	5
**Figure S4.** COSY spectrum of compound **1**.	6
**Figure S5.** HSQC spectrum of compound **1**.	7
**Figure S6.** HMBC spectrum of compound **1**.	8
**Figure S7.** HR-MS spectra of compound **1**.	9
**Figure S8.** ^{1}H NMR spectrum of compound **2**.	10
**Figure S9.** ^{13}C NMR spectrum of compound **2**.	11
**Figure S10.** DEPT135 spectrum of compound **2**.	12
**Figure S11.** COSY spectrum of compound **2**.	13
**Figure S12.** HSQC spectrum of compound **2**.	14
**Figure S13.** HMBC spectrum of compound **2**.	15
**Figure S14.** HR-MS spectra of compound **2**.	16
**Figure S15.** ^{1}H NMR spectrum of compound **3.**	17
**Figure S16.** ^{13}C NMR spectrum of compound **3**.	18
**Figure S17.** DEPT135 spectrum of compound **3**.	19
**Figure S18.** COSY spectrum of compound **3**.	20
**Figure S19.** HSQC spectrum of compound **3**.	21
**Figure S20.** HMBC spectrum of compound **3**.	22
**Figure S21.** HR-MS spectrum (Positive mode) of compound **3**.	23
**Figure S22.** ^{1}H NMR spectrum of compound **4.**	24
**Figure S23.** ^{13}C NMR spectrum of compound **4**.	25
**Figure S24.** DEPT135 spectrum of compound **4**.	26
**Figure S25.** COSY spectrum of compound **4**.	27
**Figure S26.** HSQC spectrum of compound **4**.	28
**Figure S27.** HMBC spectrum of compound **4**.	29
**Figure S28.** HR-MS spectra of compound **4**.	30
**Figure S29.** Three dimensional molecular binding models of; (**a**) Co-crystallized ligand (colored light pink) in the active site of COX-2; (**b**) Docked molecule **3** (colored green) to the active site of COX-2; (**c**) Docked molecule **4** (colored cyan) to the active site of COX-2; (**d**) Co-crystallized ligand (Arachidonic acid, colored light pink) in the active site of 5-LOX; (**e**) Simultaneously docked molecules **1A** and **1B** (colored yellow) to the active site of 5-LOX; (**f**) Co-crystallized ligand (colored light pink) in the active site of BchE.	33
**Table S1.** PDB codes of the crystal structures and grid box coordinates for the enzymes used in the docking study.	31
**Table S2.** A list of H-bonding interactions of compounds (**1A**/**1B** and **3-4**) against COX-2, 5-LOX, and BchE enzymes obtained by AutoDock Vina 1.2.3 compared to the interactions of the co-crystallized ligands.	32
**References**	34

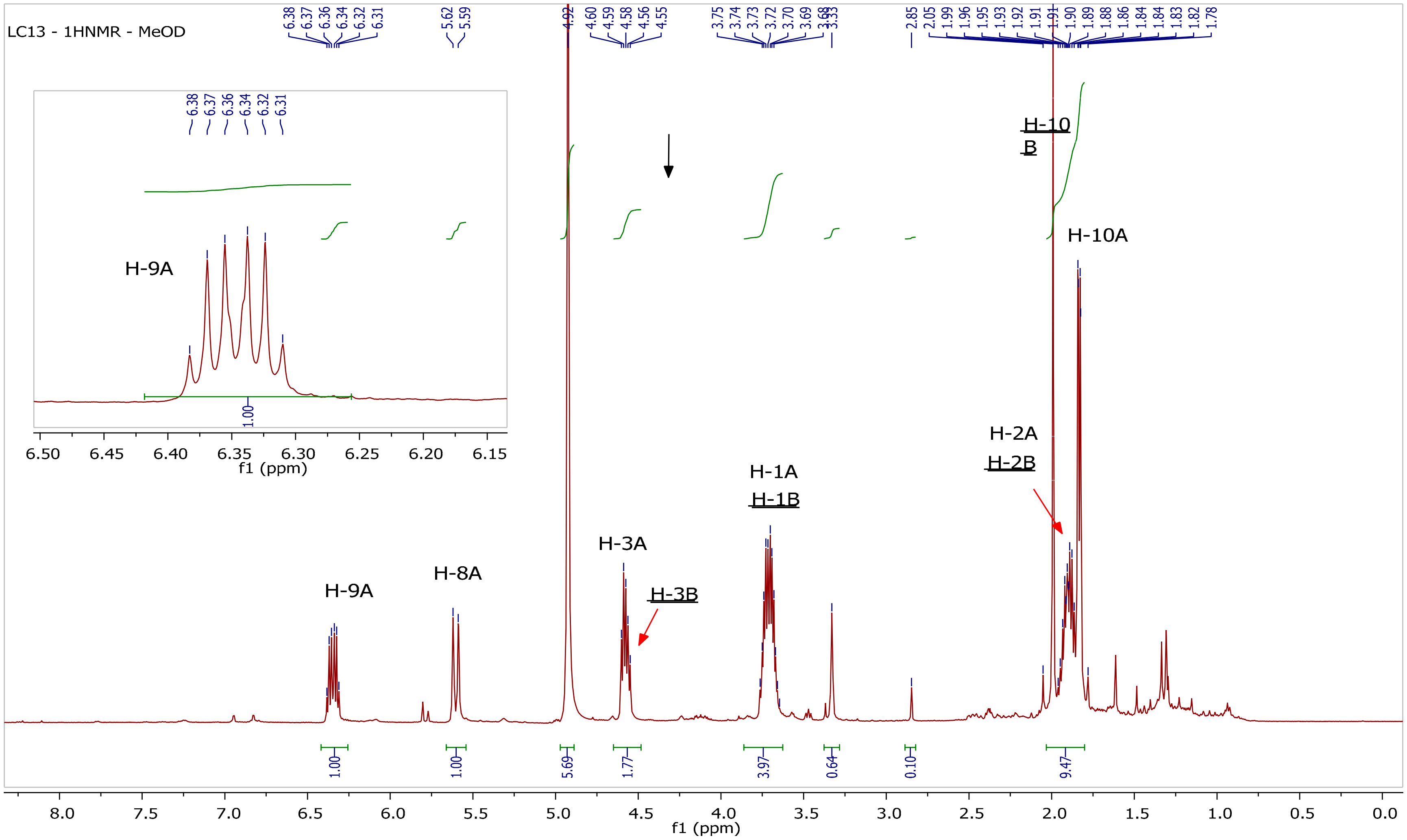

Figure S1. ^{1}H NMR spectrum of compound **1** (CD_3OD, 500 MHz).

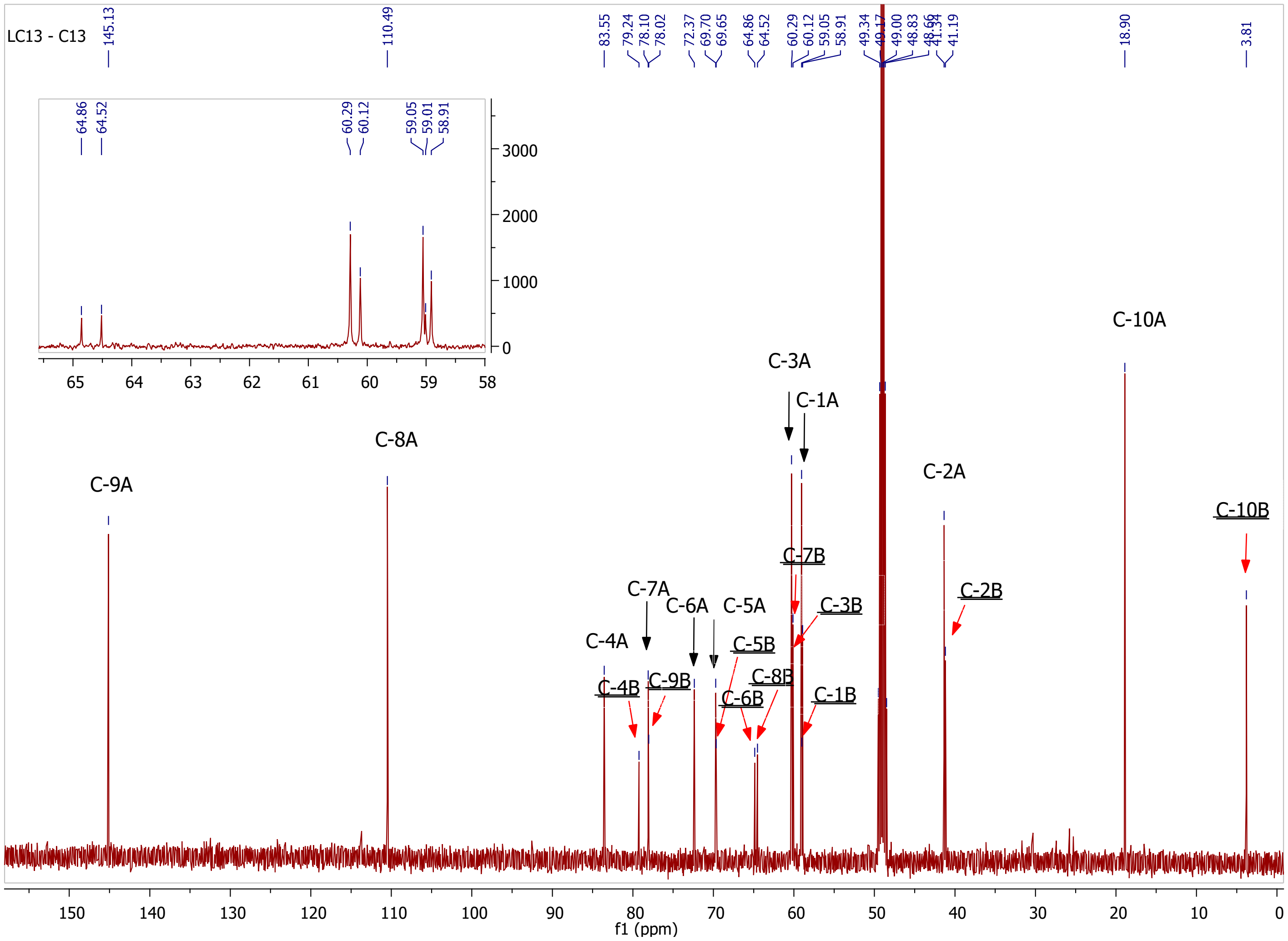

Figure S2. ^{13}C NMR spectrum of compound **1** (CD_3OD, 125 MHz).

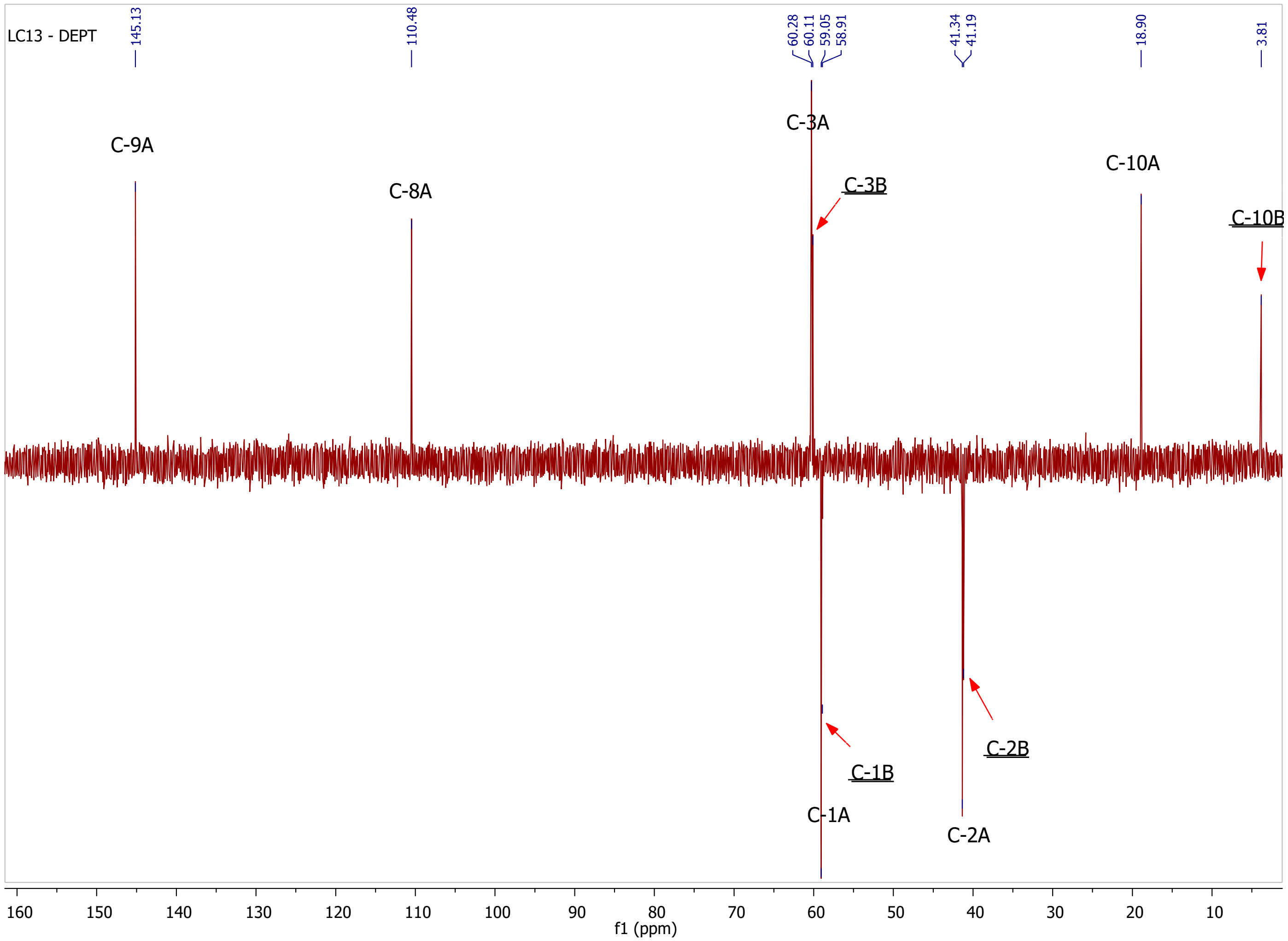

Figure S3. DEPT135 spectrum of compound **1**.

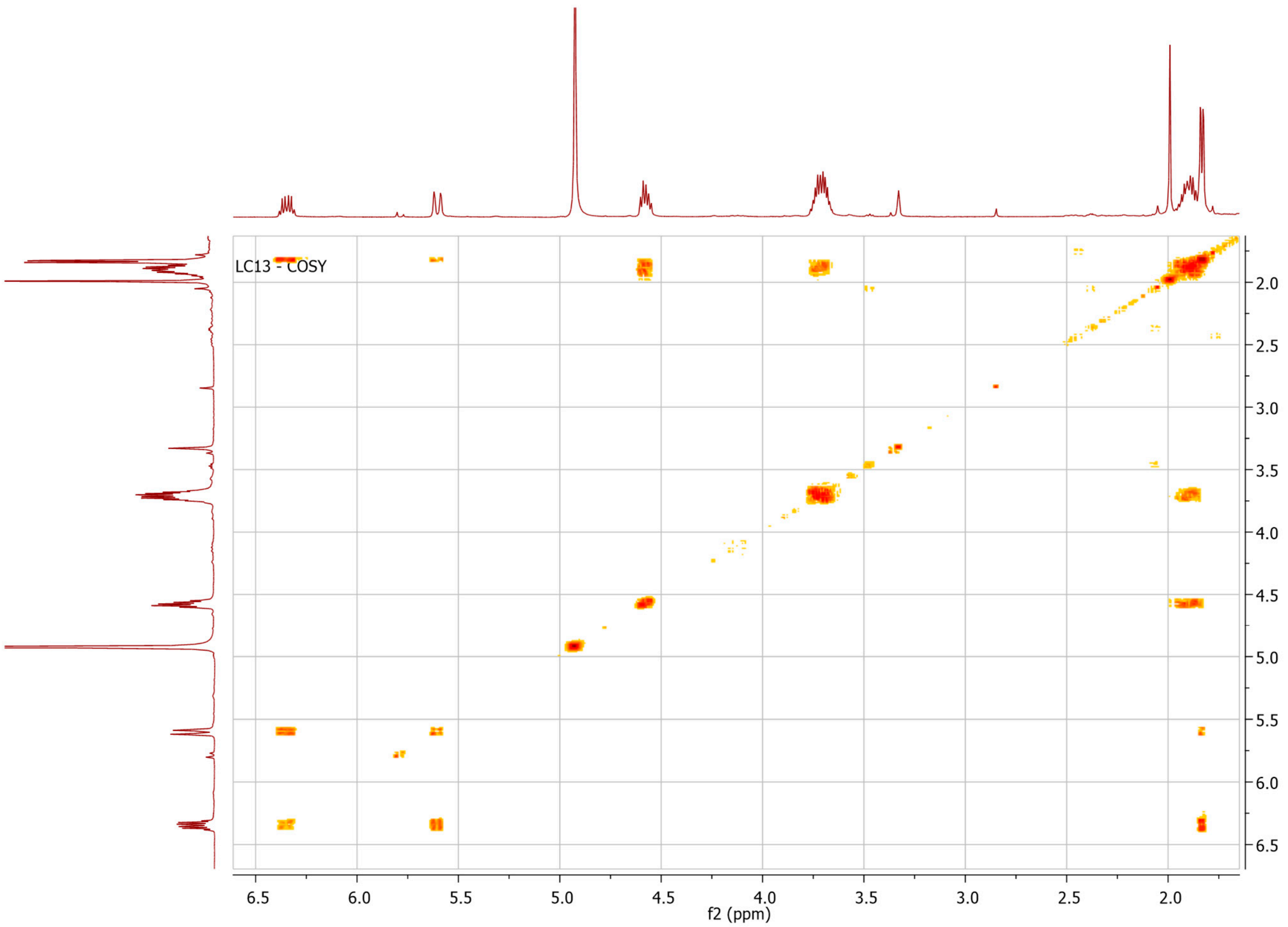

Figure S4. COSY spectrum of compound **1**.

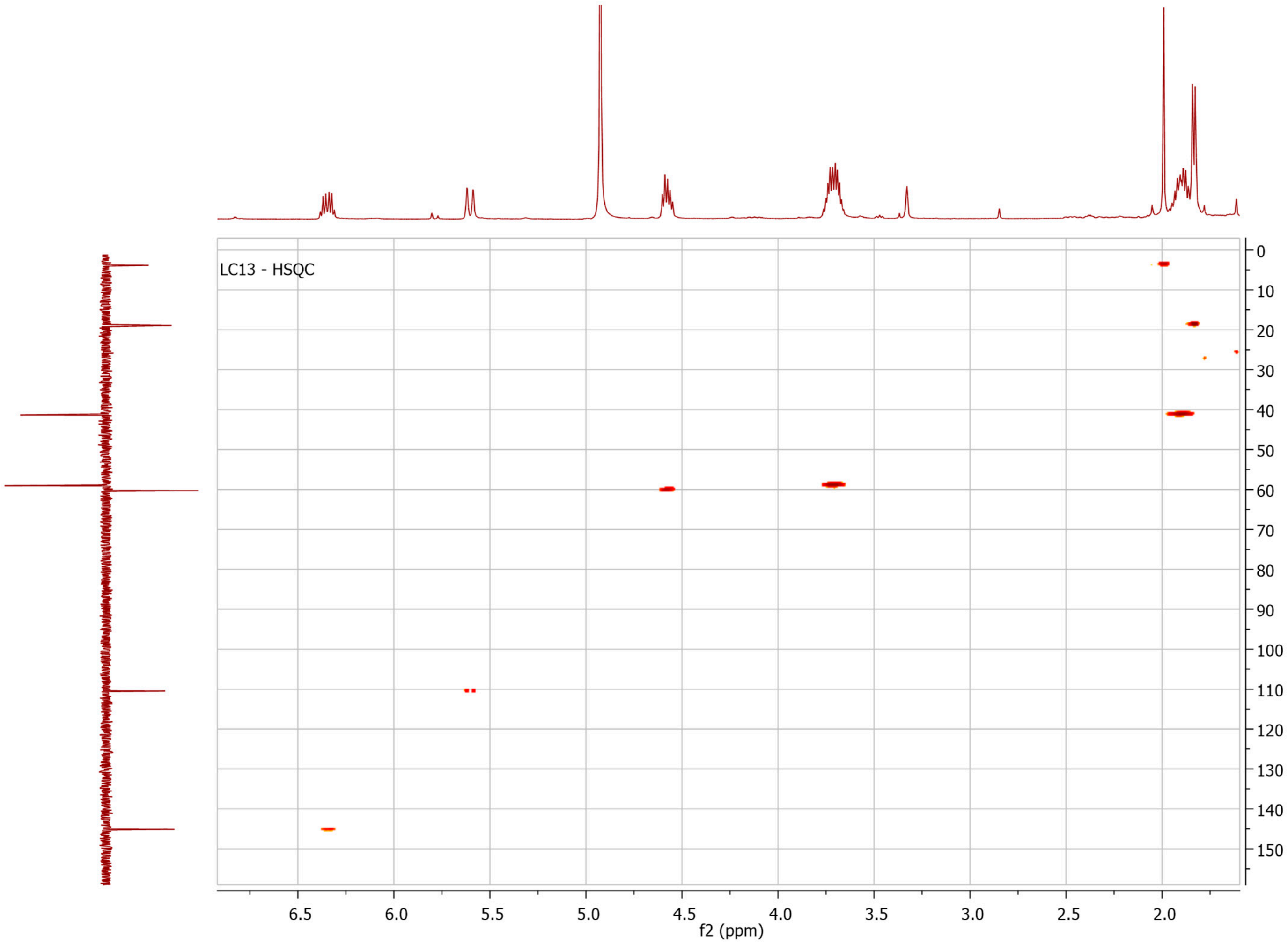

Figure S5. HSQC spectrum of compound **1**.

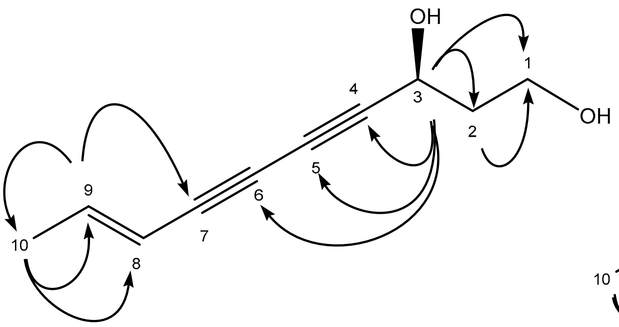

1A

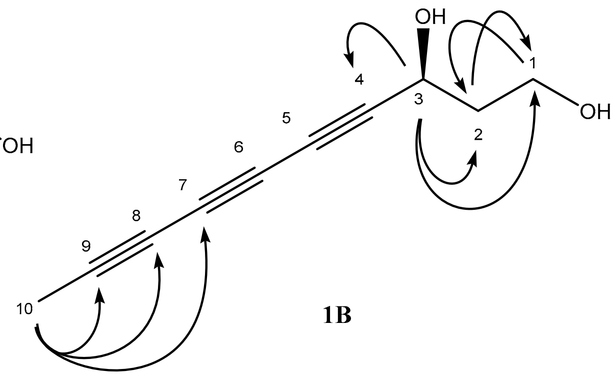

1B

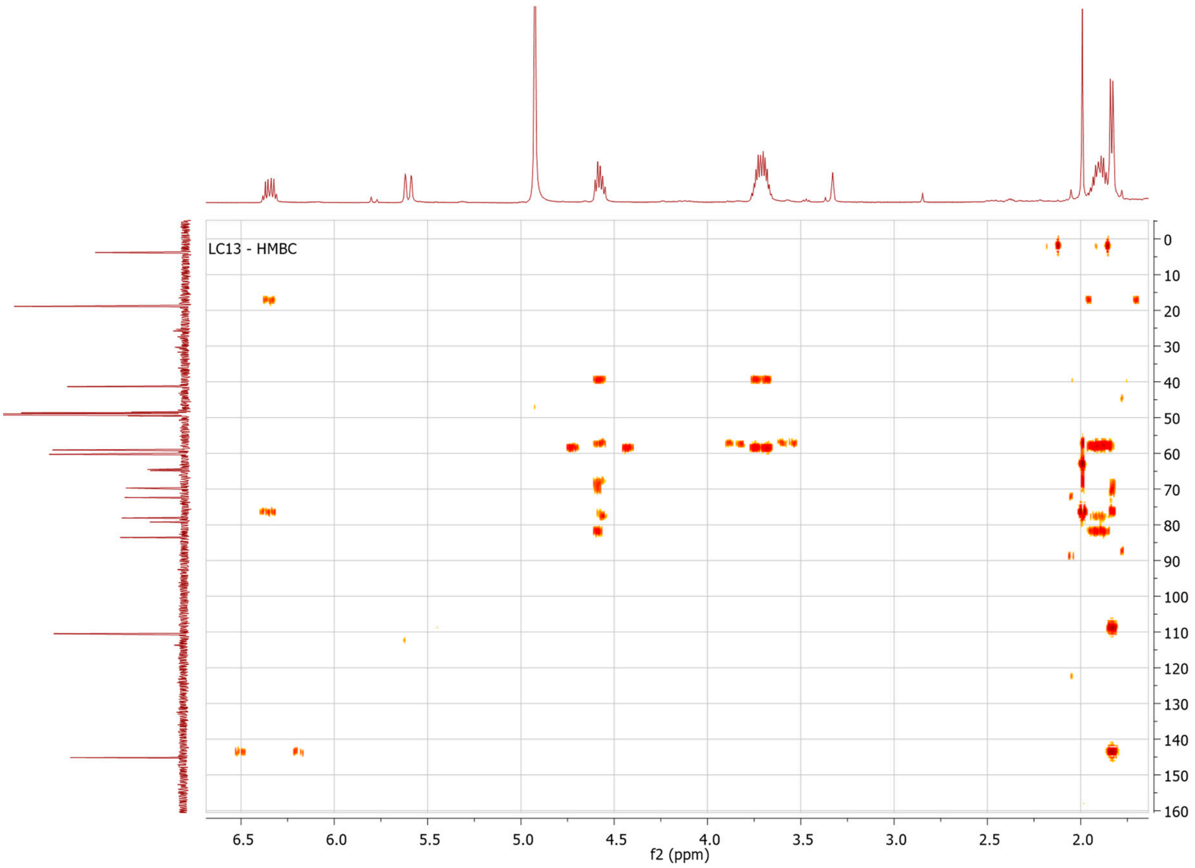

Figure S6. HMBC spectrum of compound **1**.

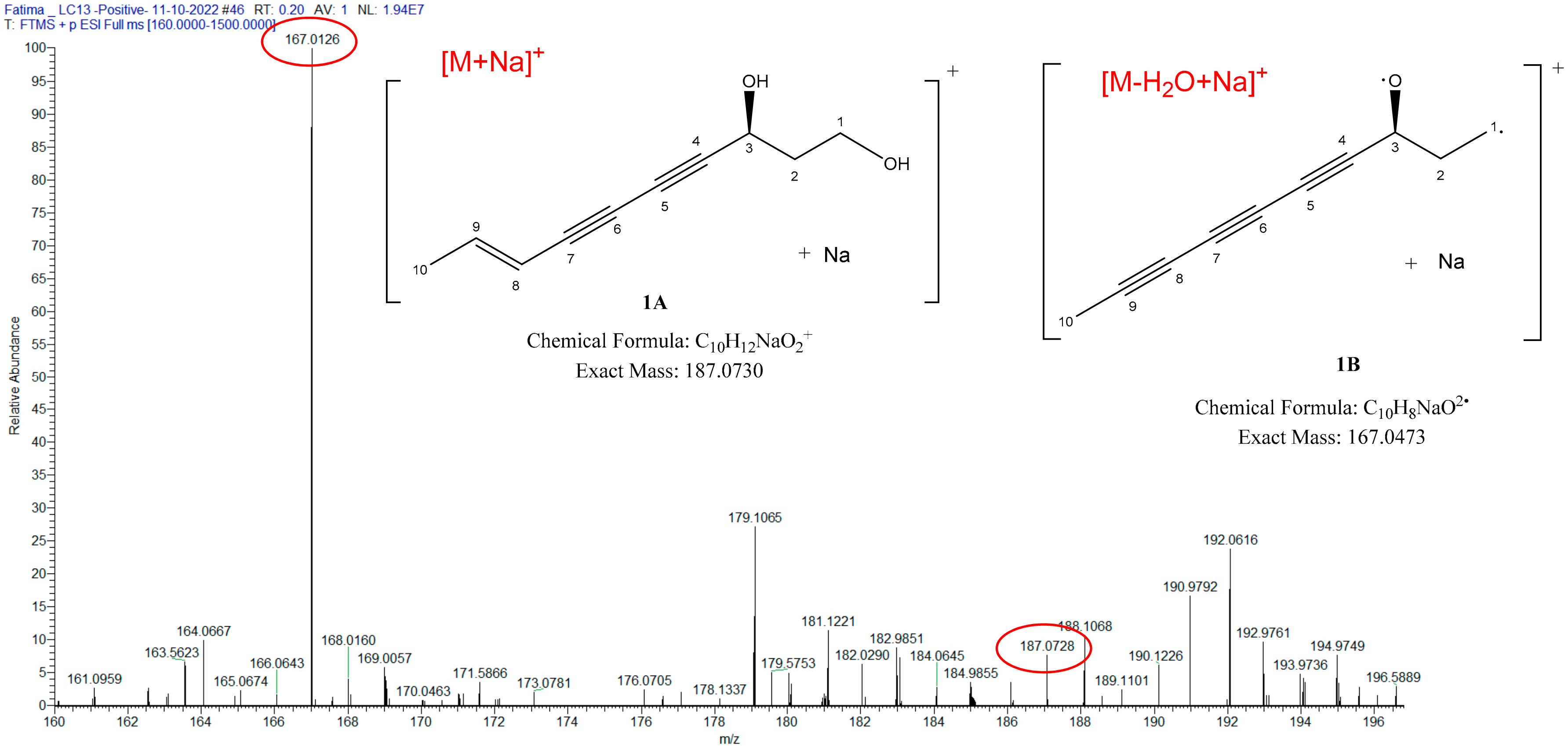

Figure S7. HR-MS (Positive mode) of compound **1**. It showed a pseudo molecular ion peaks of the mixture components at m/z 167.0126 for [M-H2O+Na]$^+$ (Calcd. 167.0473) for **1B** and 187.0728 for [M+Na]$^+$ (Calcd. 187.0730) for **1A**.

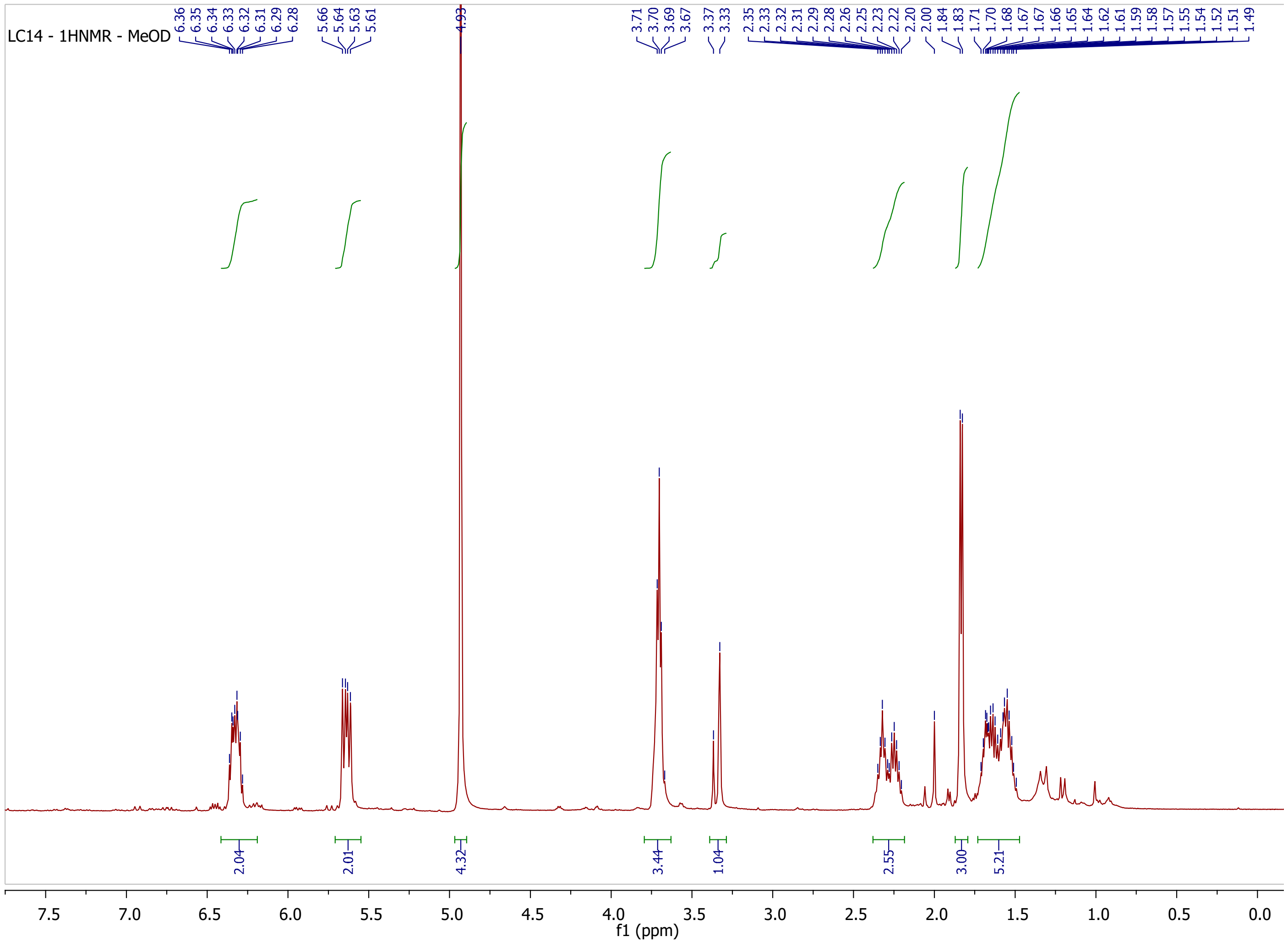

Figure S8. ^{1}H NMR spectrum of compound **2** (CD_3OD, 500 MHz).

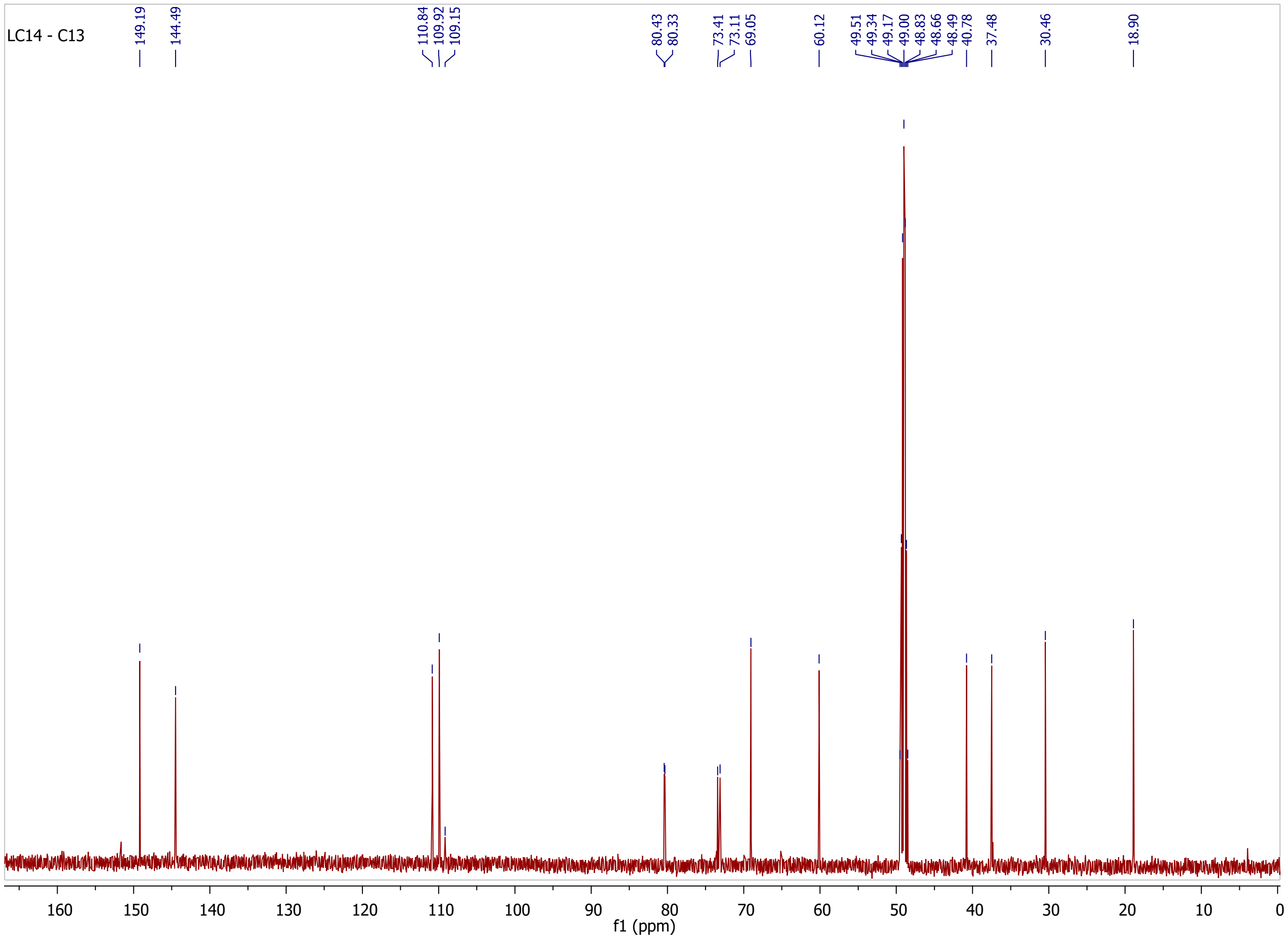

Figure S9. ^{13}C NMR spectrum of compound **2** (CD_3OD, 125 MHz).

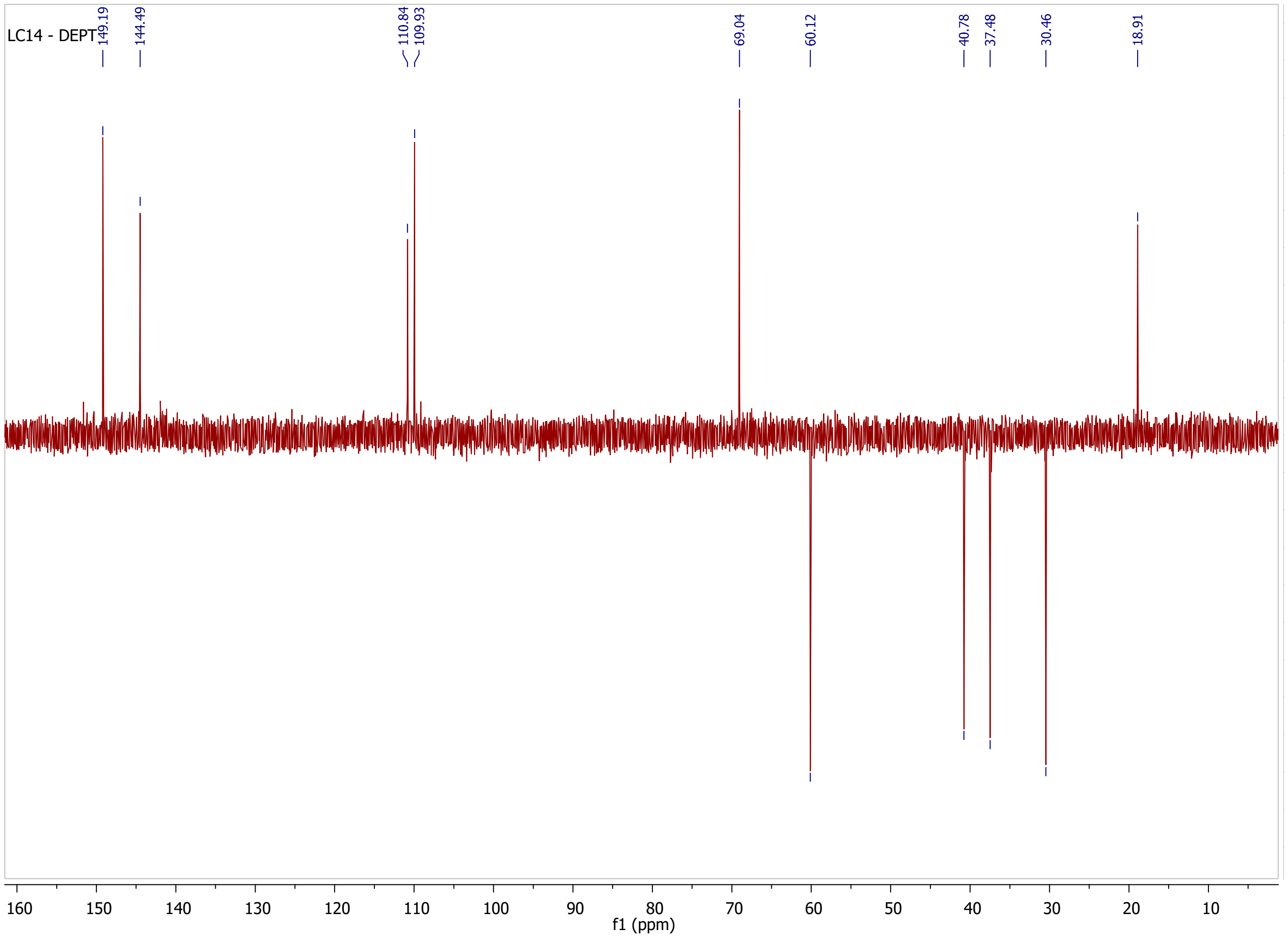

Figure S10. DEPT135 spectrum of compound **2**.

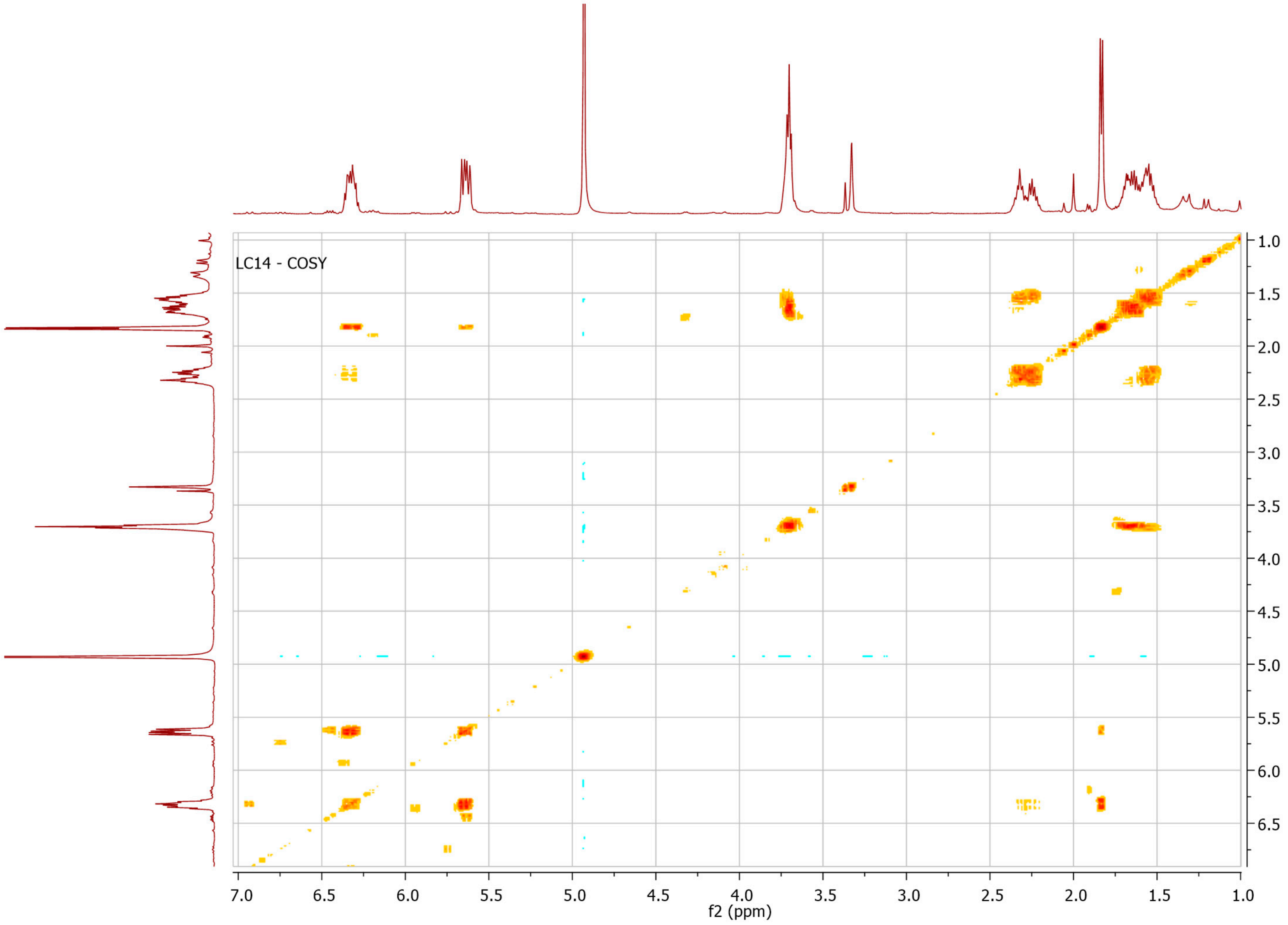

Figure S11. COSY spectrum of compound **2**.

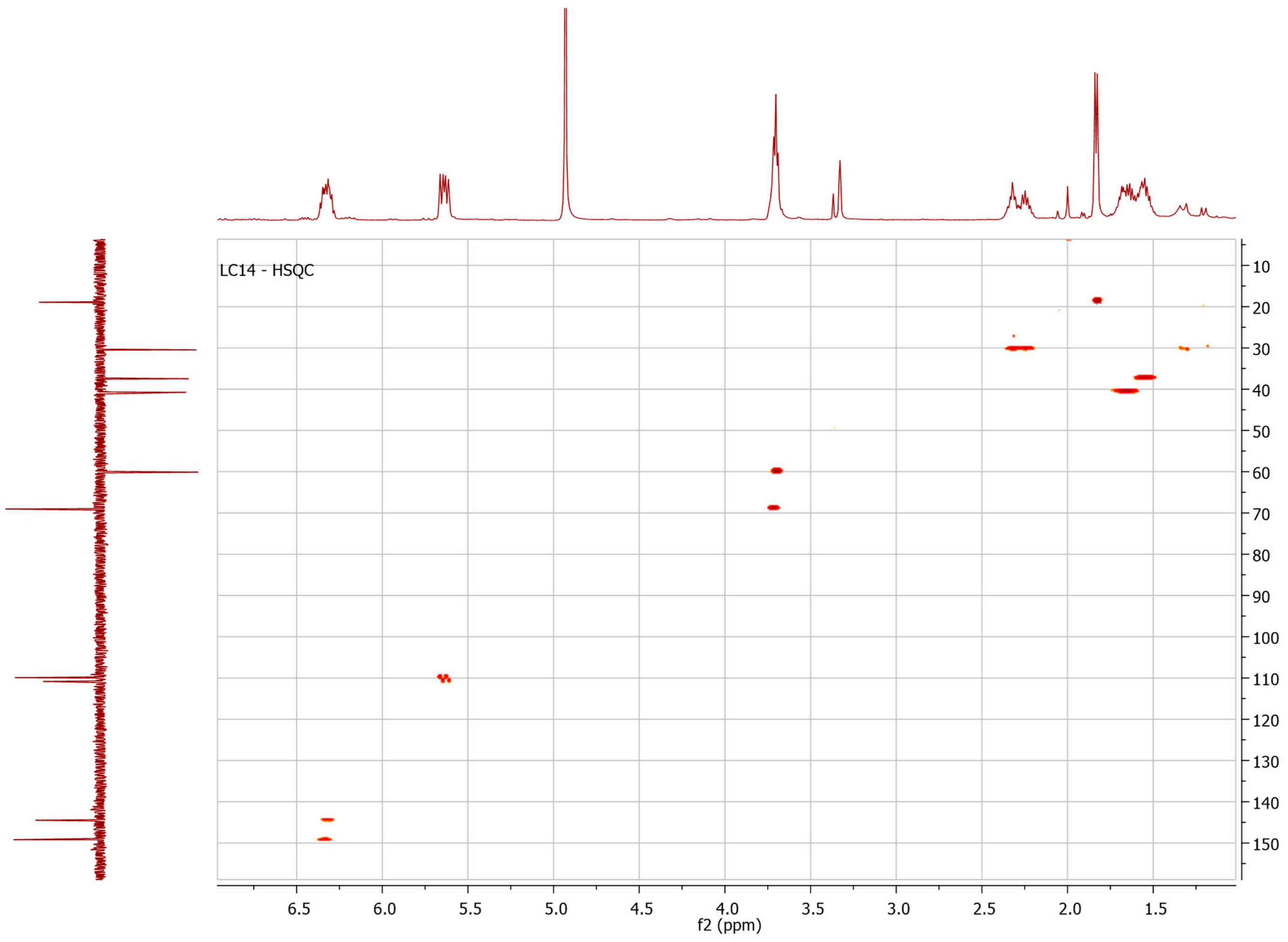

Figure S12. HSQC spectrum of compound **2**.

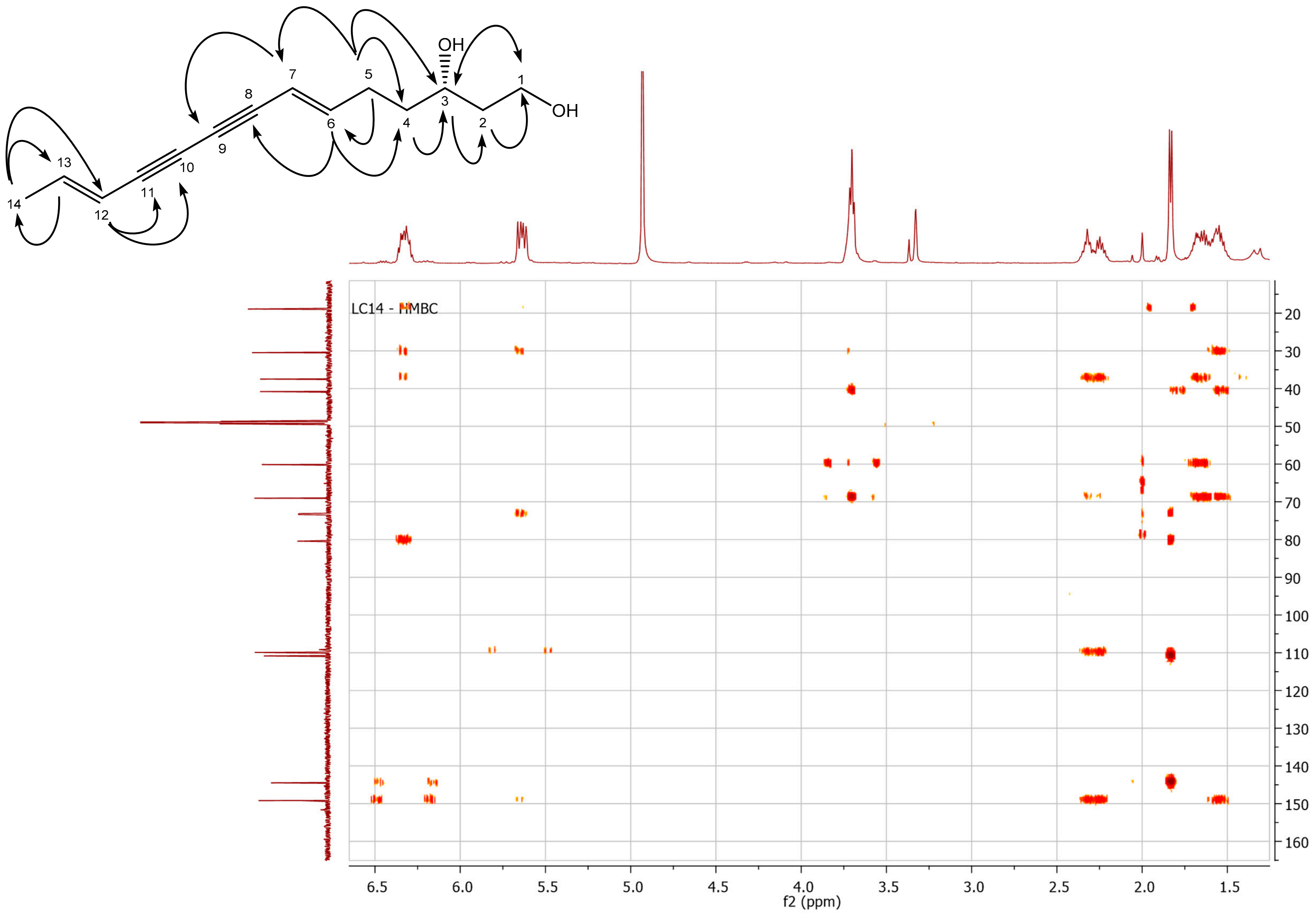

Figure S13. HMBC spectrum of compound **2**.

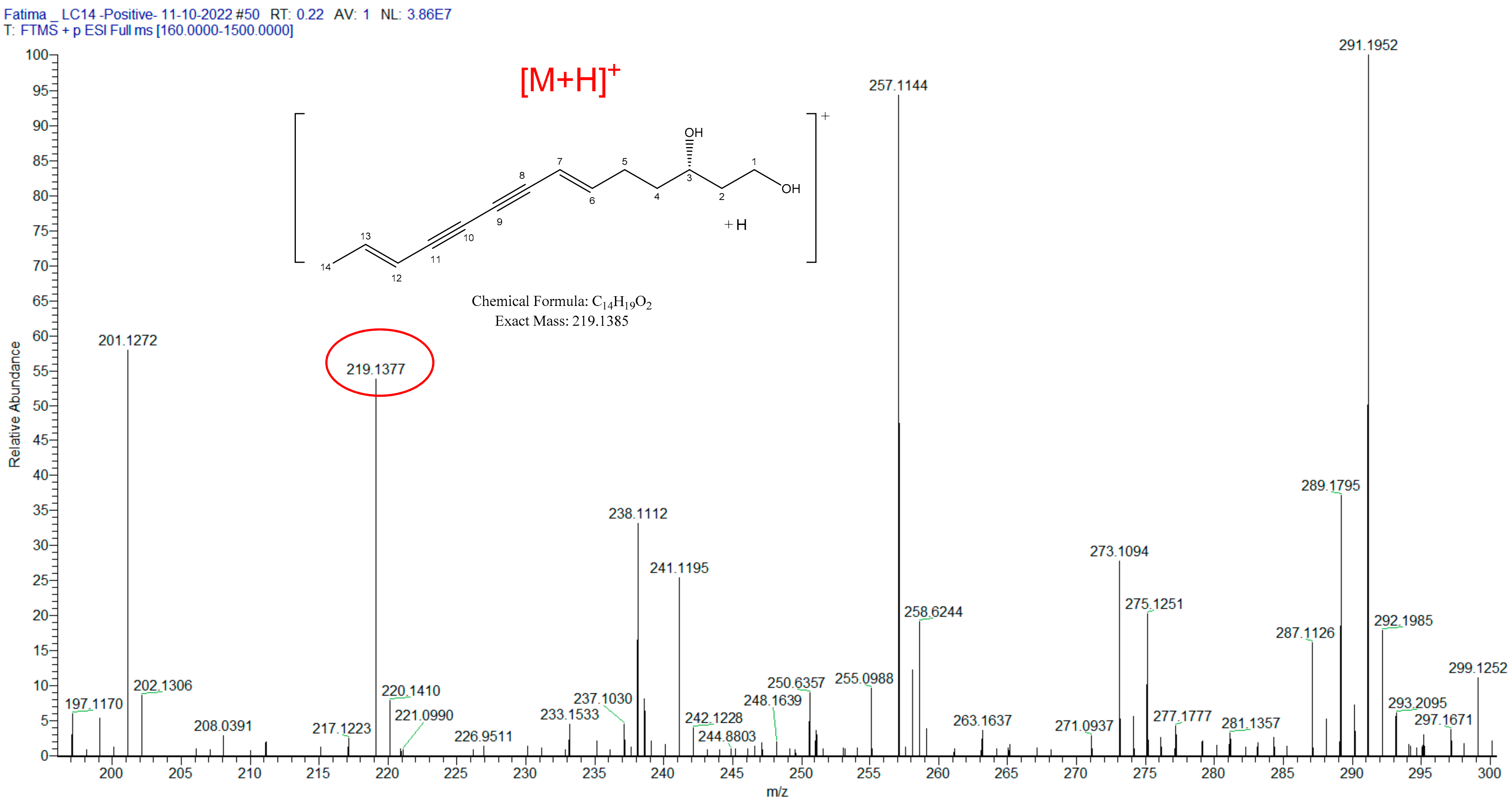

Figure S14. HR-MS (Positive mode) of compound **2**. It showed a peak of 219.1377 of $[M+Na]^+$ (Calcd. at 219.1385).

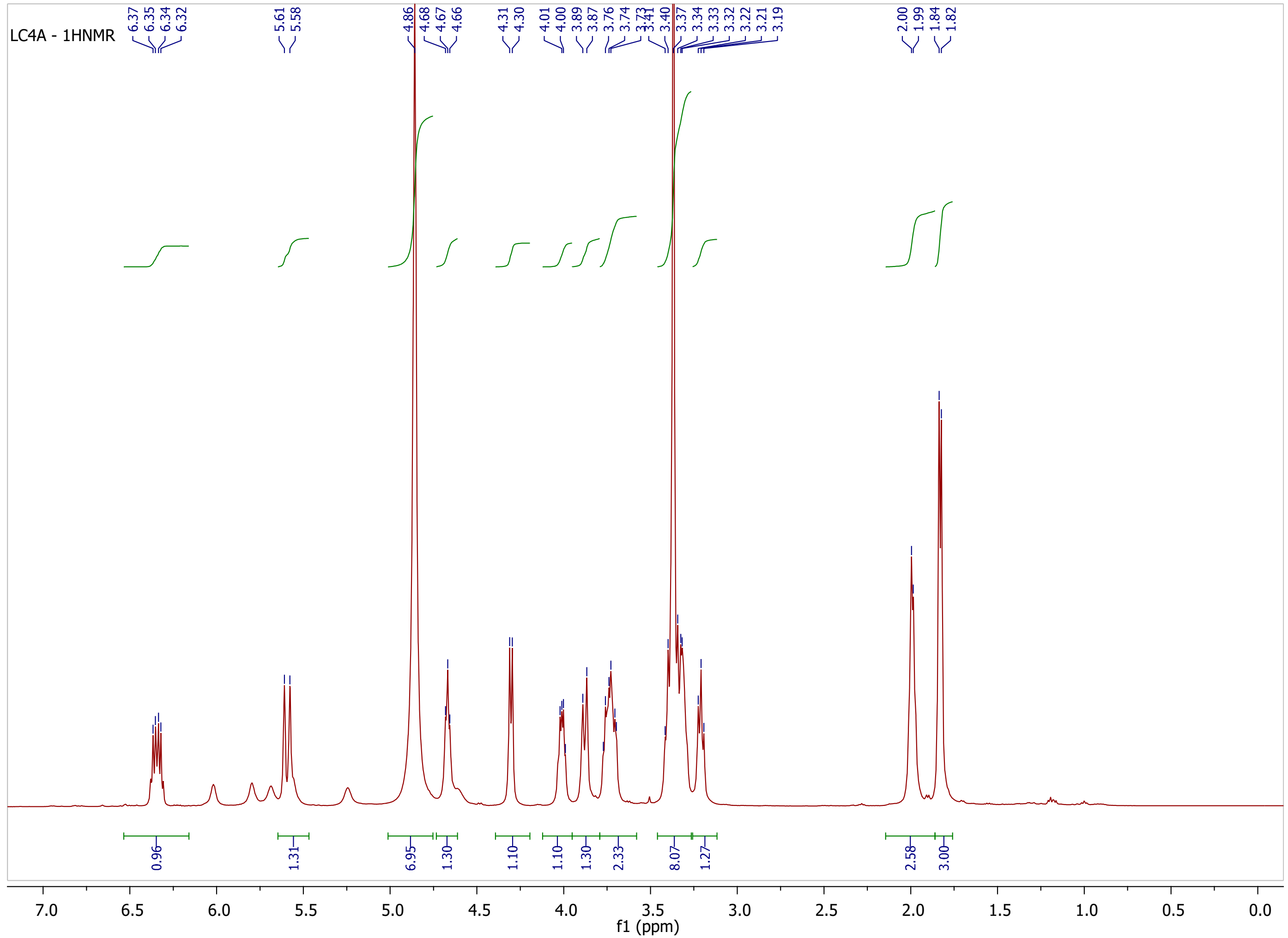

Figure S15. ^{1}H NMR spectrum of compound **3** (CD_3OD, 500 MHz).

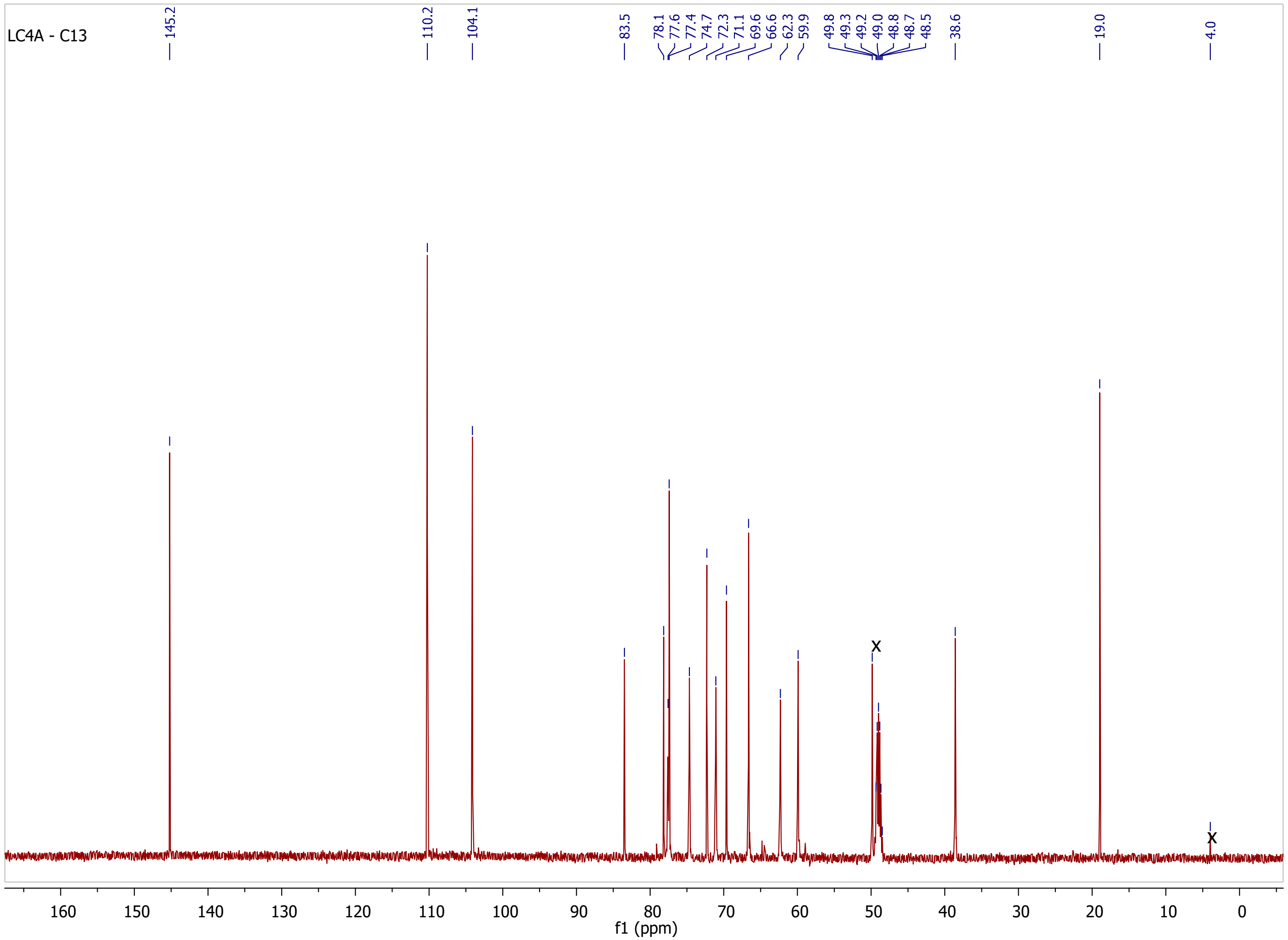

Figure S16. ^{13}C NMR spectrum of compound **3** (CD_3OD, 125 MHz).

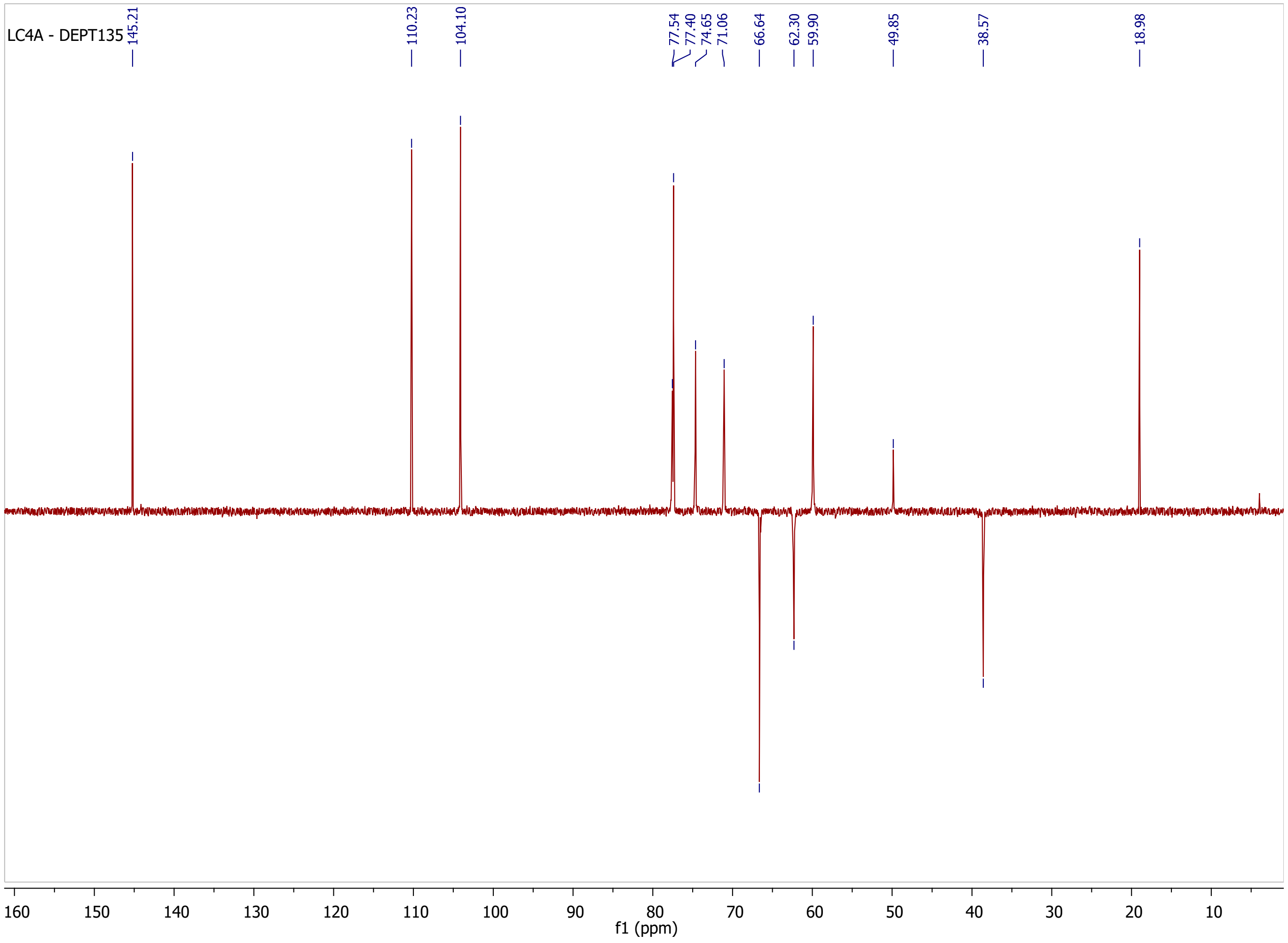

Figure S17. DEPT135 spectrum of compound **3**.

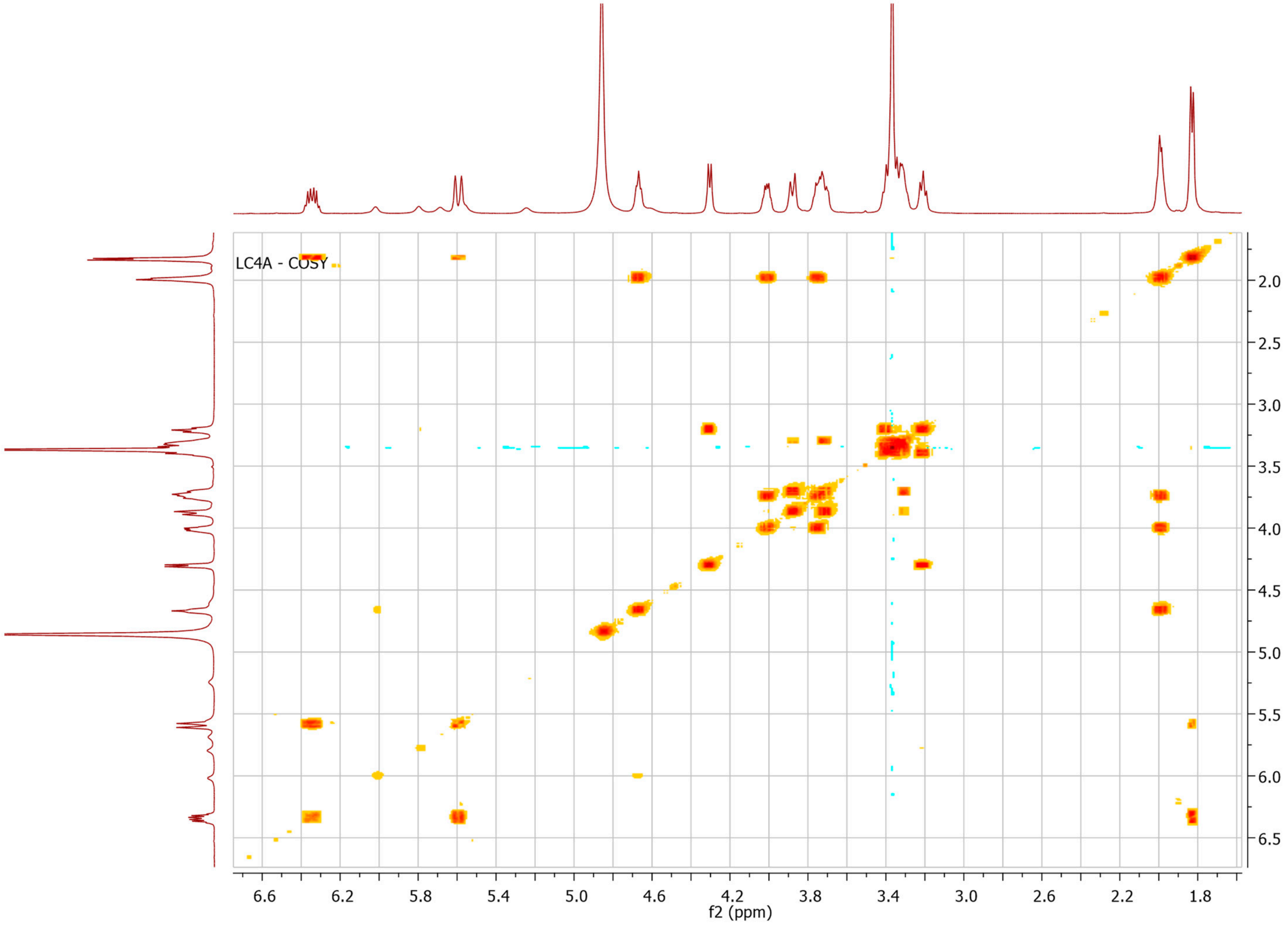

Figure S18. COSY spectrum of compound **3**.

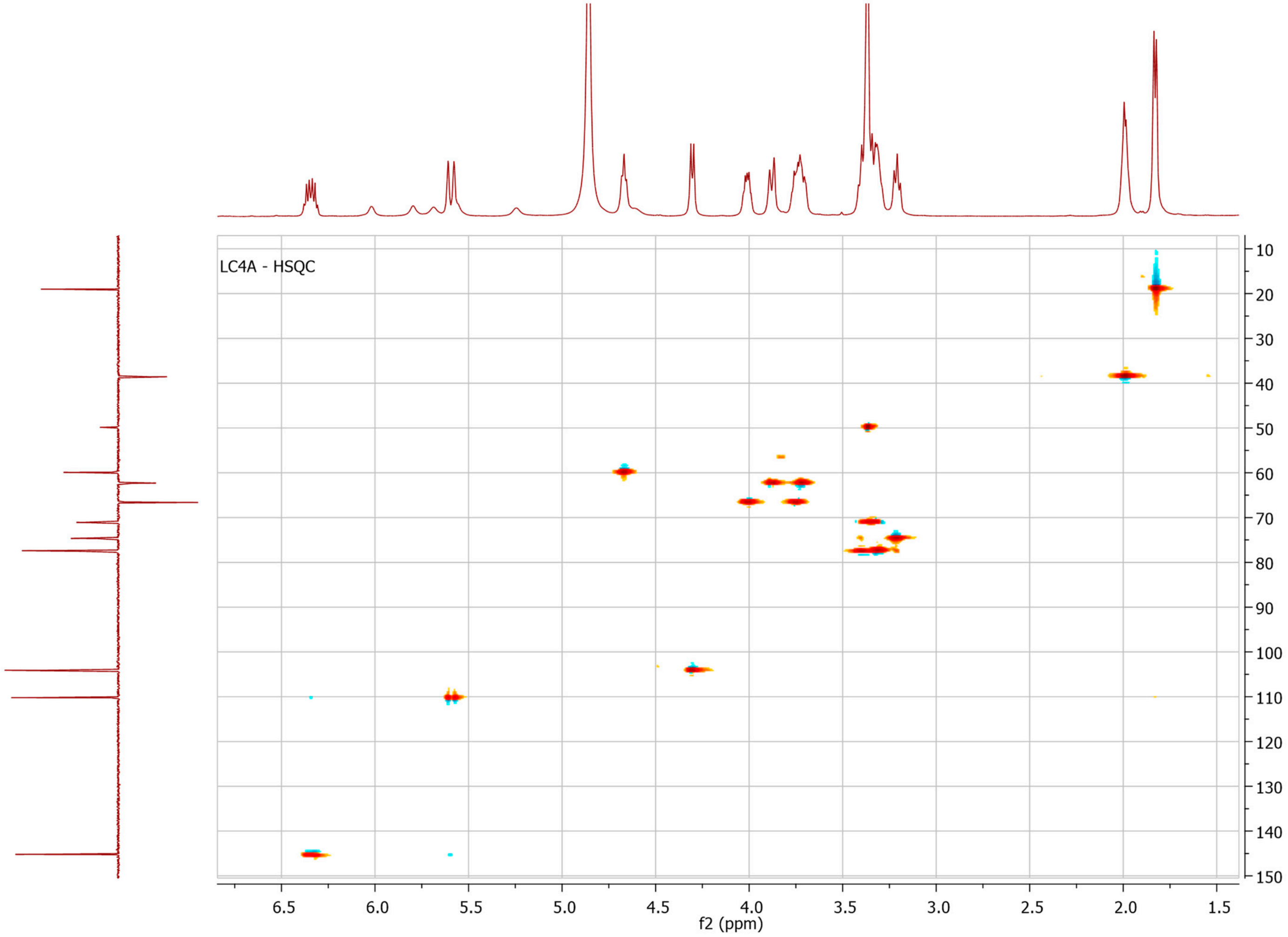

Figure S19. HSQC spectrum of compound **3**.

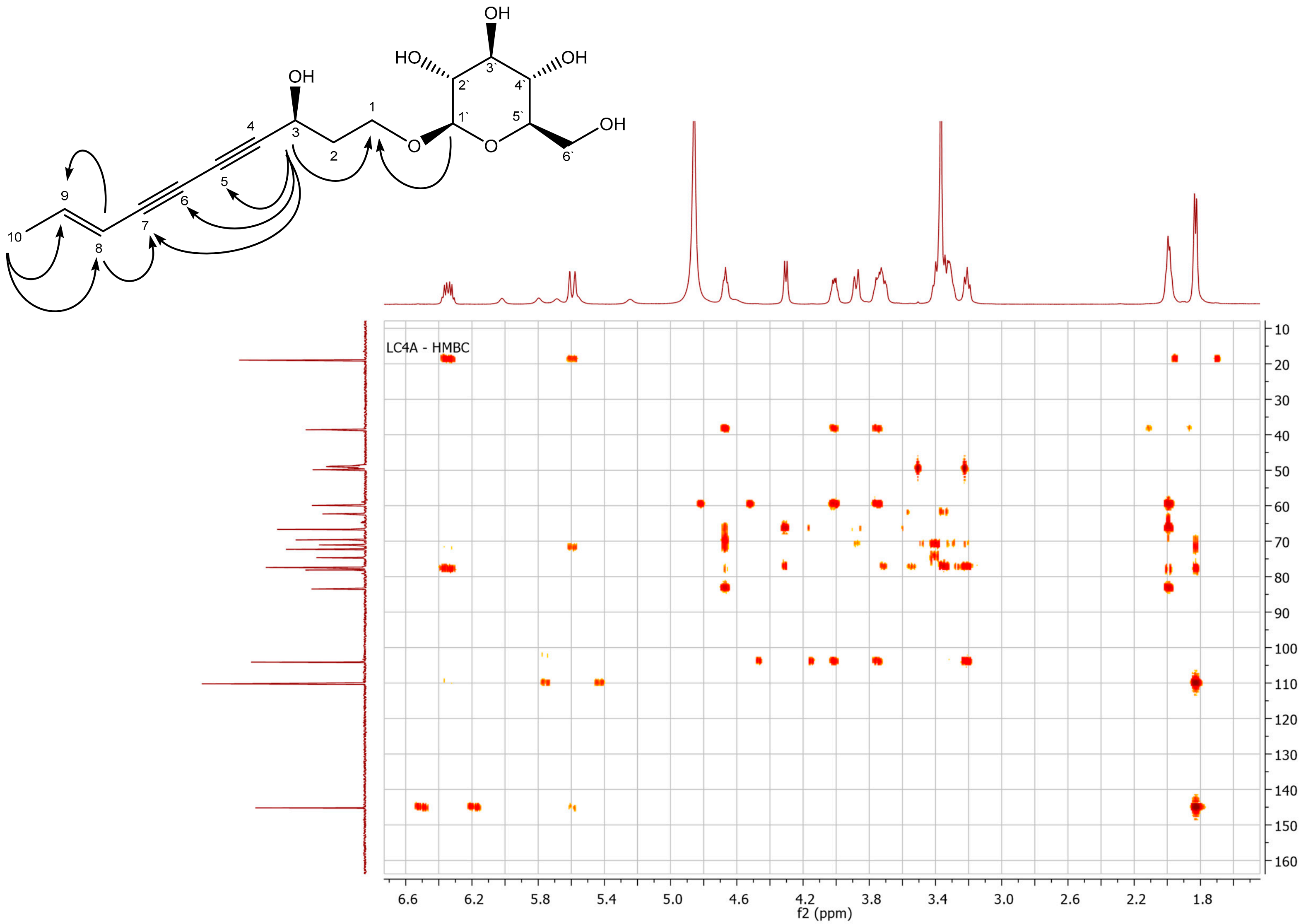

Figure S20. HMBC spectrum of compound **3**.

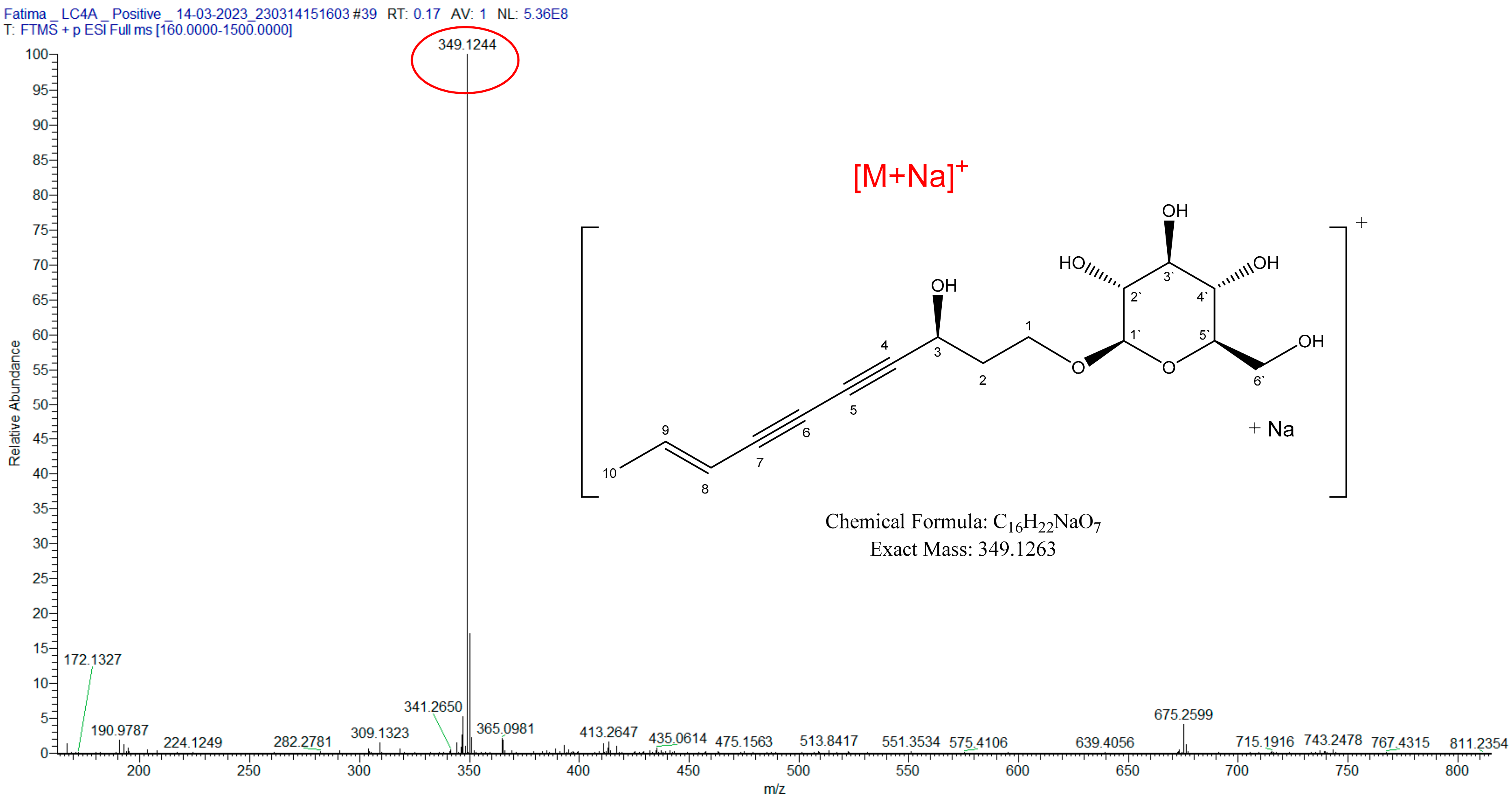

Figure S21. HR-MS spectrum (Positive mode) of compound **3**. It showed a pseudo molecular ion peak at 349.1244 (Calcd. at 349.1263).

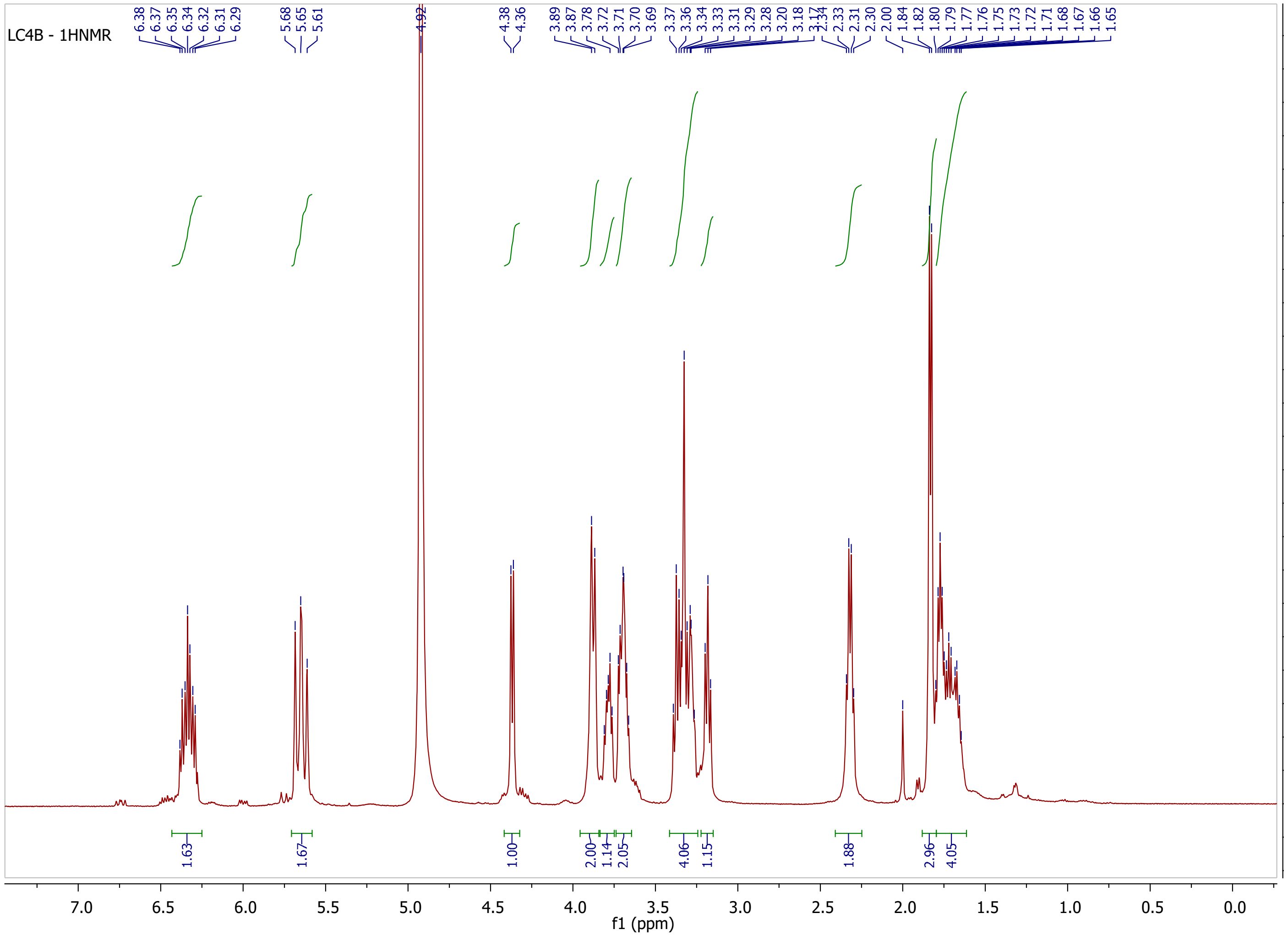

Figure S22. ^{1}H NMR spectrum of compound **4** (CD_3OD, 500 MHz).

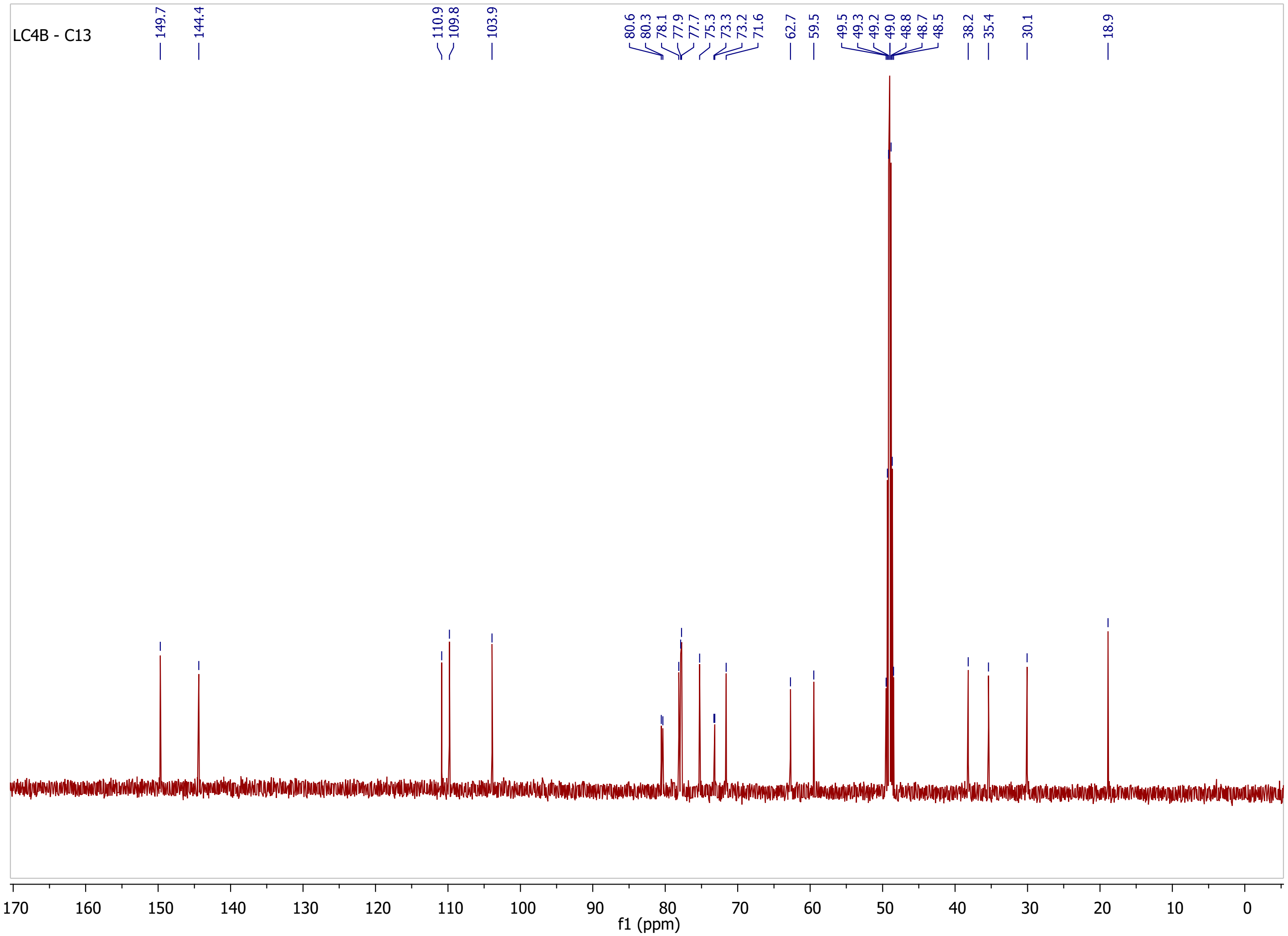

Figure S23. ^{13}C NMR spectrum of compound **4** (CD_3OD, 125 MHz).

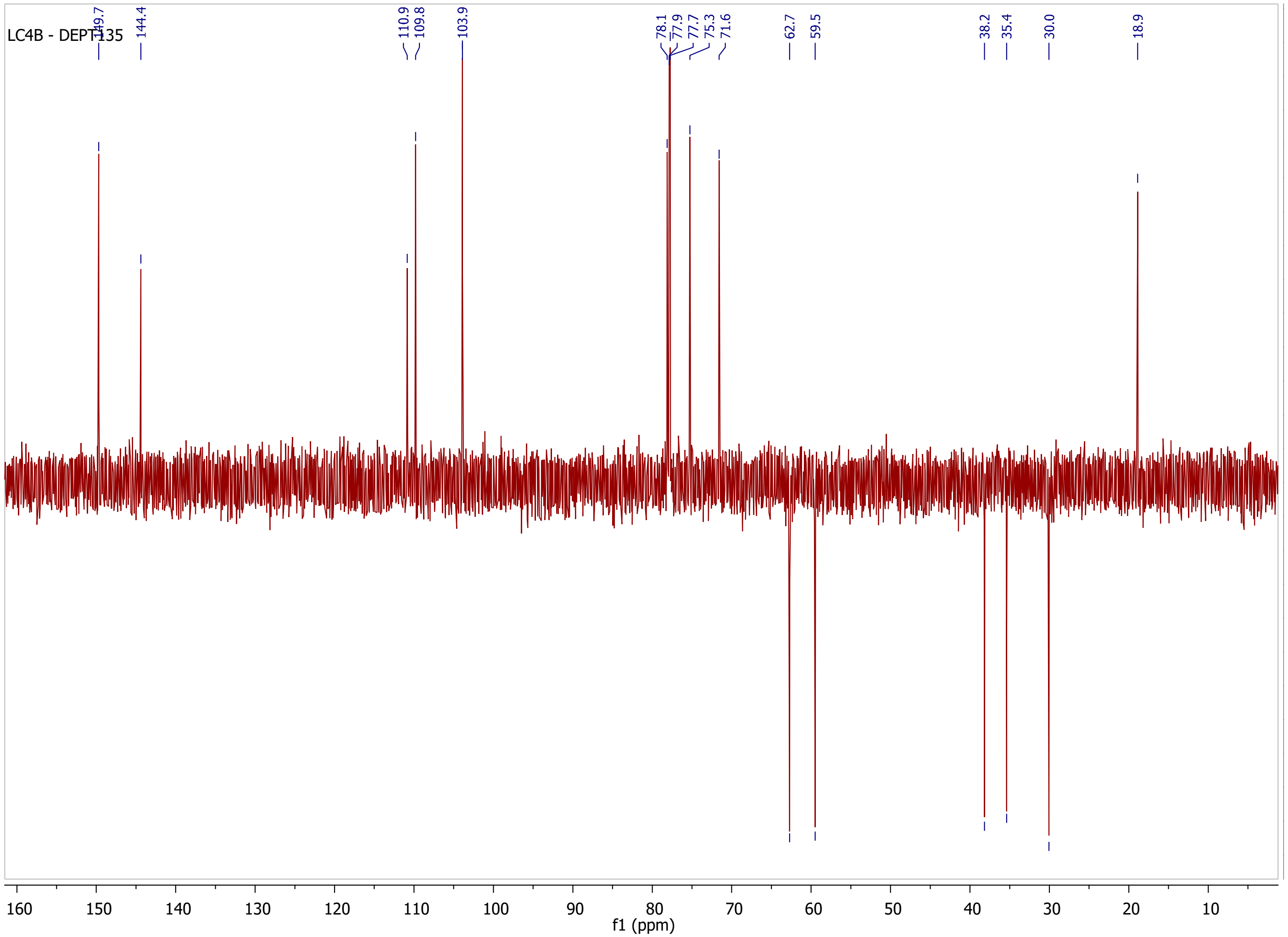

Figure S24. DEPT135 spectrum of compound **4**.

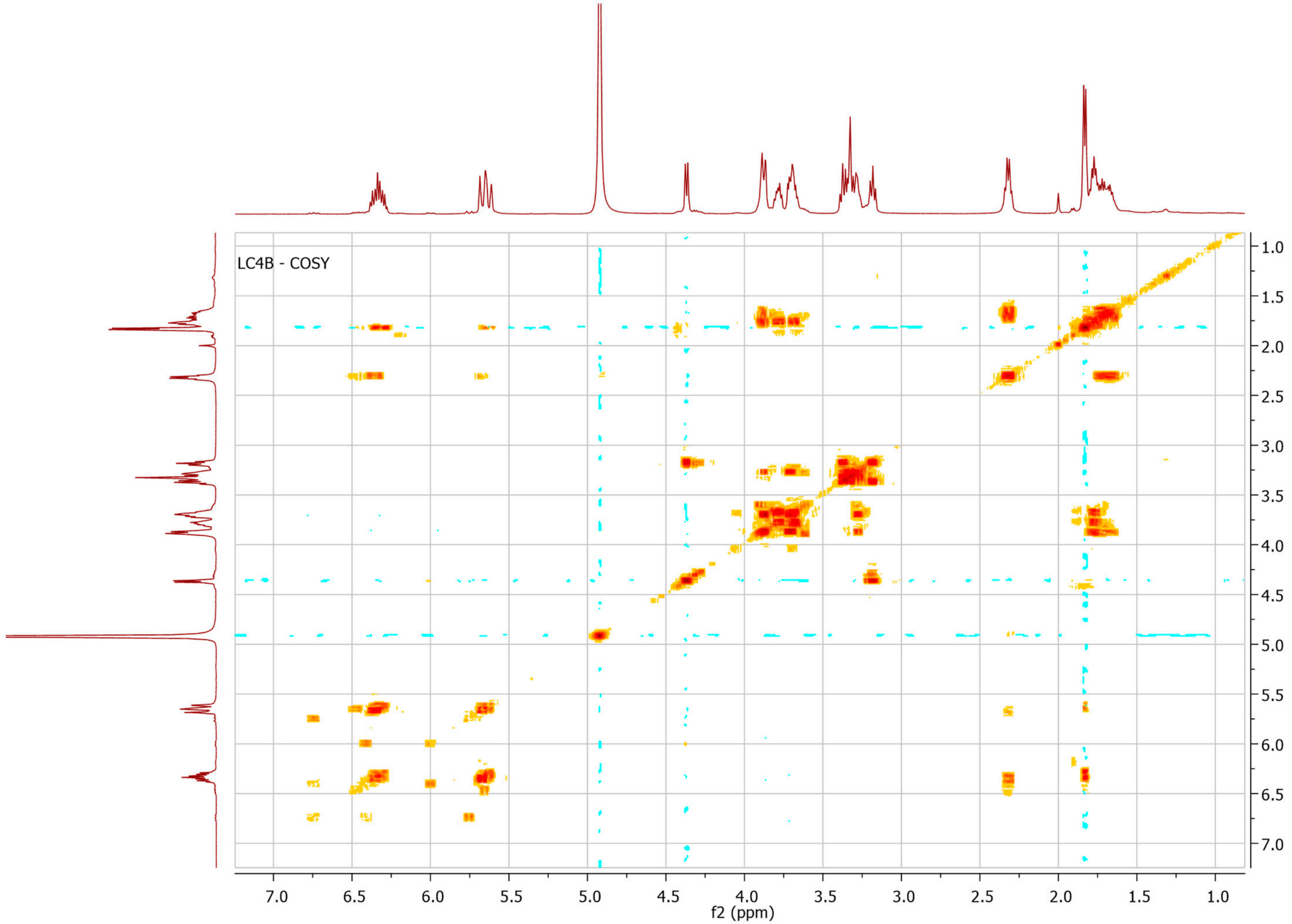

Figure S25. COSY spectrum of compound **4**.

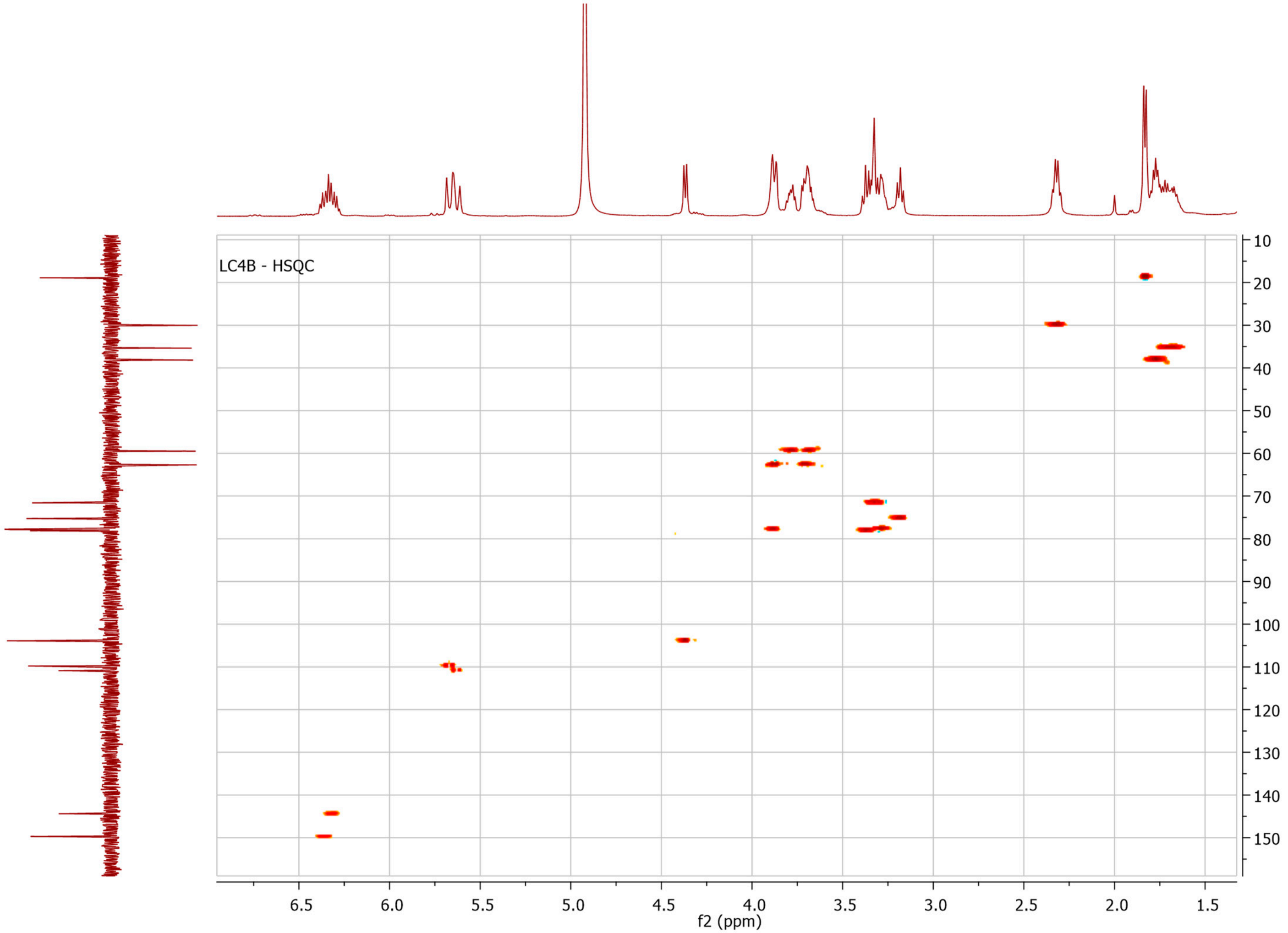

Figure S26. HSQC spectrum of compound **4**.

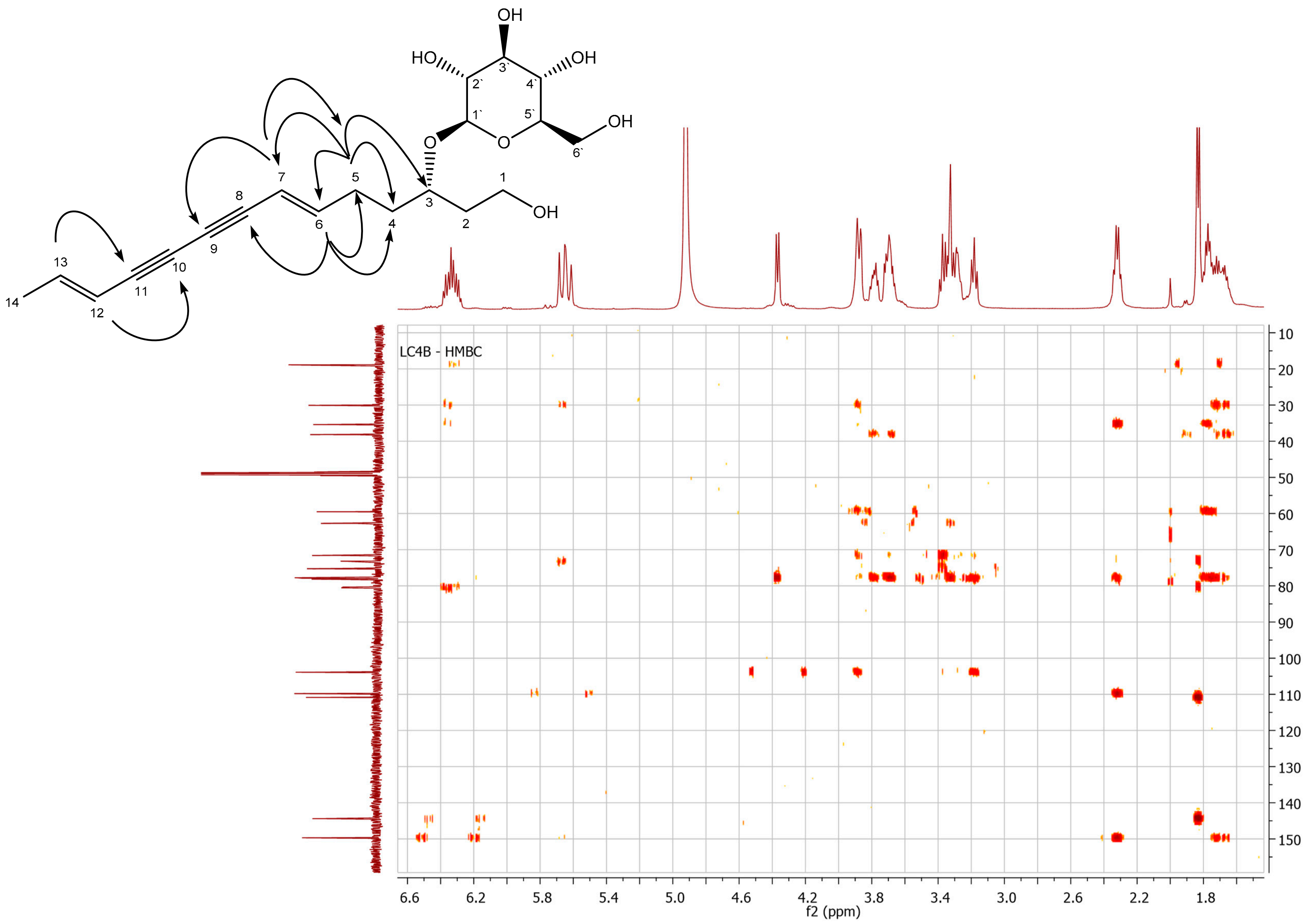

Figure S27. HMBC spectrum of compound **4**.

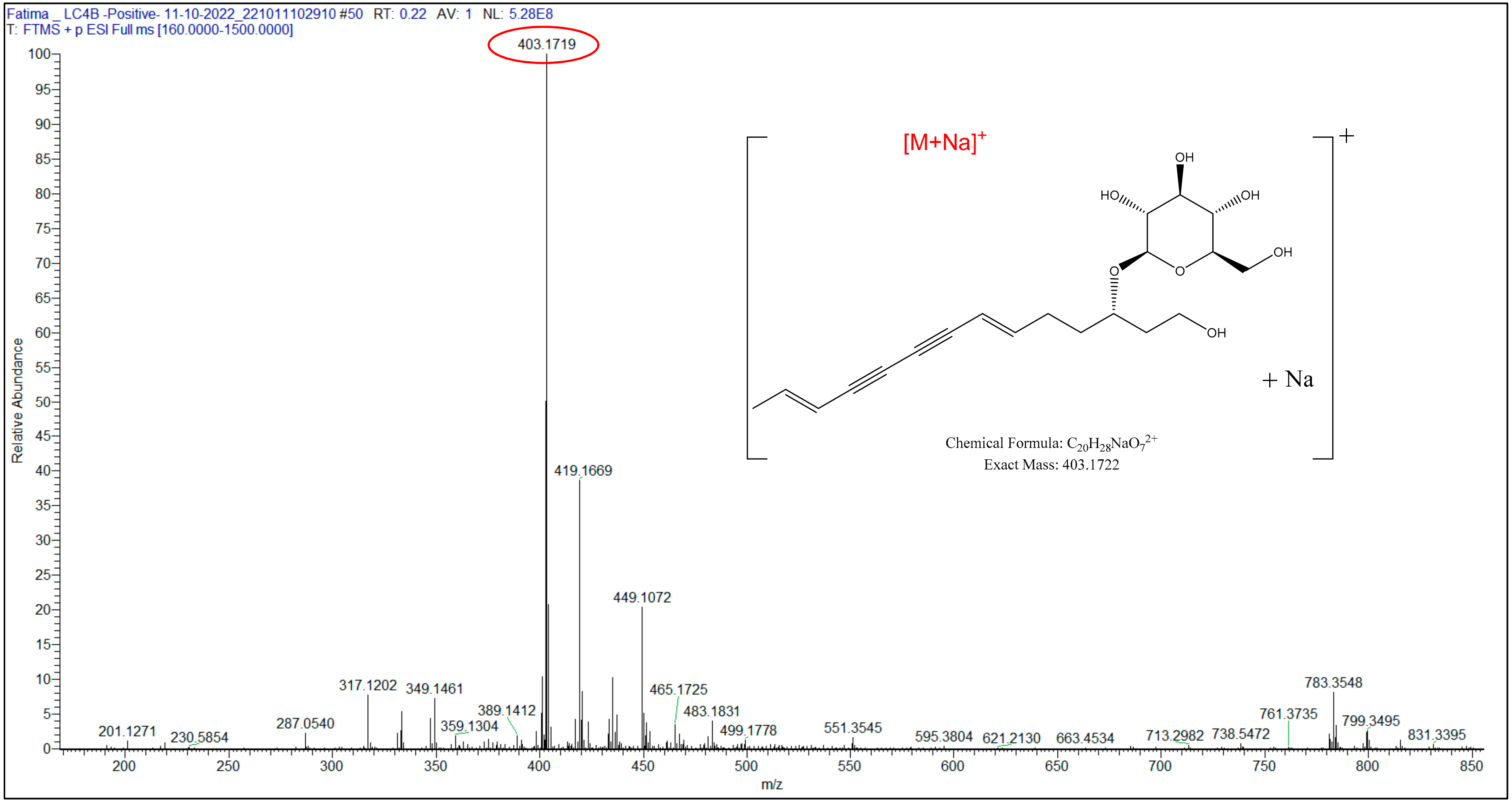

Figure S28. HR-MS spectrum (Positive mode) of compound **4**. It showed a pseudo molecular ion peak at 403.1719 (Calcd. at 403.1722).

Table S1. PDB codes of the crystal structures and grid box coordinates for the enzymes used in the docking study.

Enzymes	PDB codes	Grid box X, Y and Z coordinates	Co-crystallized ligand (Reference inhibitor)
**cyclooxygenase-II (COX-2)**	5IkV	166.123 185.599 and 191.004	**FLF:** flufenamic Acid (2-[[3-(trifluoromethyl)phenyl]amino] benzoic acid)
**5-lipooxygenase (5-LOX)**	3V99	19.448 -76.699 and -34.290	**ACD:** arachidonic acid
**butyrylcholine esterase (BchE)**	6ESY	5.576 -12.575 and -13.512	**TFX:** thioflavine T (2-[4-(dimethylamino)phenyl]-3,6-dimethyl-1,3-benzothiazol-3-ium)

Table S2. A list of H-bonding interactions of compounds (**1A/1B** and **3-4**) against COX-2, 5-LOX, and BchE enzymes obtained by AutoDock Vina 1.2.3 compared to the interactions of the co-crystallized ligands.

**Compound**	**Hydrogen bonding interactions**		
	**Cyclooxygenase-II (COX-2)**	**5-lipooxygenase (5-LOX)**	**Butyrylcholine esterase (BchE)**
**1A/1B (simultaneous docking)**	Arg-120, Glu-524	Lys-173, Tyr-558, Phe-555, Asn-554	Tyr-128
**3**	Trp-387, Thr-206	His-372, His-367, His-550, Asn-554	Tyr-128, His-438, Trp-82
**4**	Asp-347, Gln-350, His-351, Tyr-355, phe-580	Val-175, Asp-176, Ala-606	Trp-82, Trp-430, Gly-116, Gly-117
**Co-crystallized ligand**	Ser-530, Tyr-385 as exhibited in Fig. S29a reported by Orlando, and Malkowski [1]	Arachidonic acid was reported to form van der Waals contacts with the active site of 5-LOX, and no H-bonds are formed [2]	Two molecules of TFX are docked to the active site of BchE as presented in fig. S29f. They are reported to form aromatic stacking with Tyr-332, but no Hydrogen bonds are reported to be formed [3]

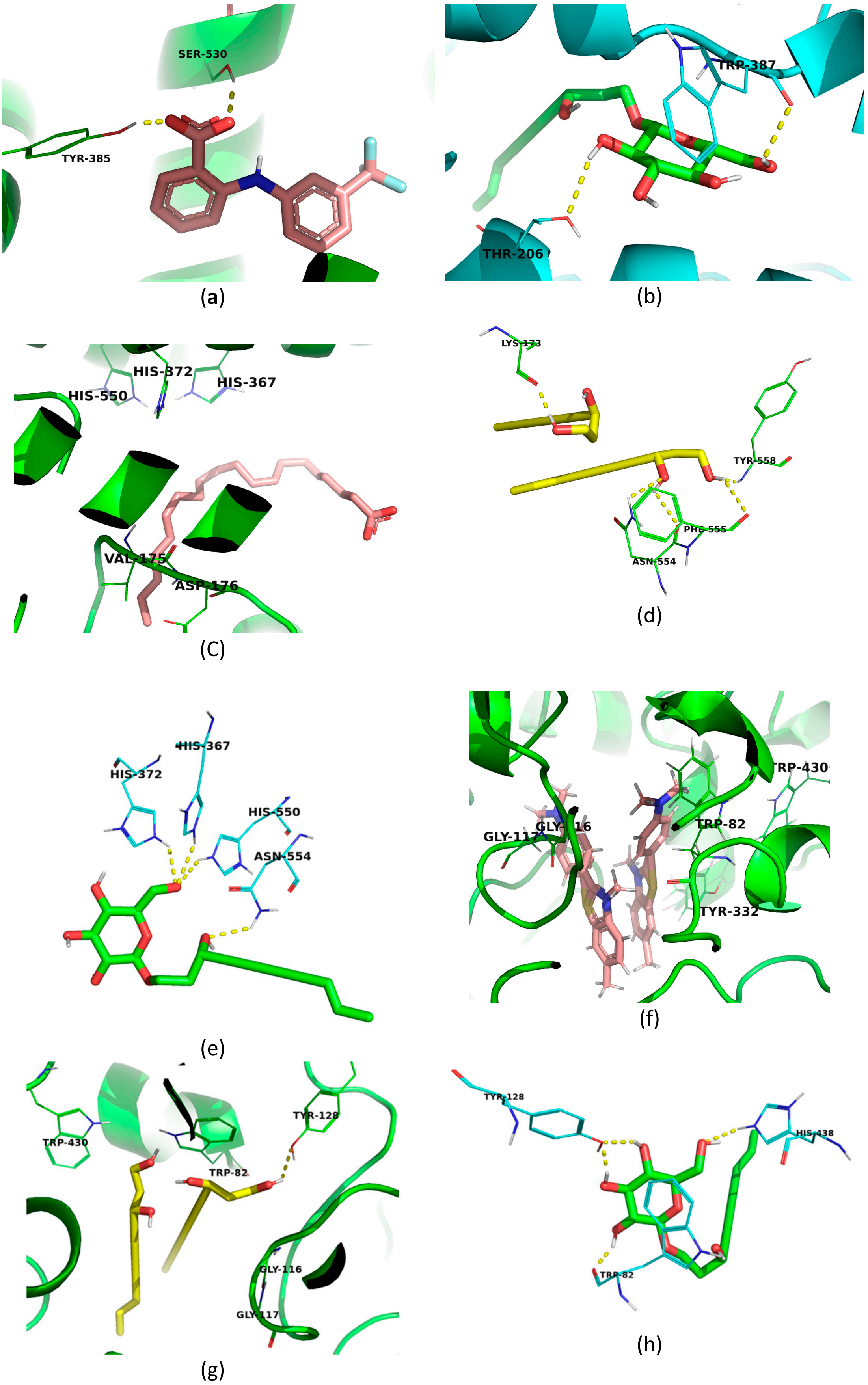

Figure S29. Three-dimensional molecular binding models of; (**a**) Co-crystallized ligand (colored light pink) in the active site of COX-2; (**b**) Docked molecule **3** (colored

green) to the active site of COX-2; (**c**) Co-crystallized ligand (Arachidonic acid, colored light pink) in the active site of 5-LOX; (**d**) Simultaneously docked molecules **1A** and **1B** (colored yellow) to the active site of 5-LOX; (**e**) Docked molecule 3 (colored green) to the active site of 5-LOX (**f**) Co-crystallized ligand (colored light pink) in the active site of BchE; g) Simultaneously docked molecules 1A and 1B (colored yellow) to the active site of BchE; (h) Docked molecule 3 (colored green) to the active site of BchE

References:

1. Orlando, B.J.; Malkowski, M.G. Substrate-selective Inhibition of Cyclooxygeanse-2 by Fenamic Acid Derivatives Is Dependent on Peroxide Tone. *J. Biol. Chem.* **2016**, *291*, 15069-15081, doi:10.1074/jbc.M116.725713.
2. Hsu, K.-C.; HuangFu, W.-C.; Lin, T.E.; Chao, M.-W.; Sung, T.-Y.; Chen, Y.-Y.; Pan, S.-L.; Lee, J.-C.; Tzou, S.-C.; Sun, C.-M.; et al. A site-moiety map and virtual screening approach for discovery of novel 5-LOX inhibitors. *Sci. Rep.* **2020**, *10*, 10510, doi:10.1038/s41598-020-67420-9.
3. Rosenberry, T.L.; Brazzolotto, X.; Macdonald, I.R.; Wandhammer, M.; Trovaslet-Leroy, M.; Darvesh, S.; Nachon, F. Comparison of the Binding of Reversible Inhibitors to Human Butyrylcholinesterase and Acetylcholinesterase: A Crystallographic, Kinetic and Calorimetric Study. *Molecules* **2017**, *22*, 2098, doi:10.3390/molecules22122098.
